# DICER regulates the expression of major satellite repeat transcripts and meiotic chromosome segregation during spermatogenesis

**DOI:** 10.1093/nar/gkaa460

**Published:** 2020-06-02

**Authors:** Ram Prakash Yadav, Juho-Antti Mäkelä, Hanna Hyssälä, Sheyla Cisneros-Montalvo, Noora Kotaja

**Affiliations:** Institute of Biomedicine, Integrative Physiology and Pharmacology Unit, University of Turku, Finland; Institute of Biomedicine, Integrative Physiology and Pharmacology Unit, University of Turku, Finland; Institute of Biomedicine, Integrative Physiology and Pharmacology Unit, University of Turku, Finland; Institute of Biomedicine, Integrative Physiology and Pharmacology Unit, University of Turku, Finland; Institute of Biomedicine, Integrative Physiology and Pharmacology Unit, University of Turku, Finland

## Abstract

Constitutive heterochromatin at the pericentric regions of chromosomes undergoes dynamic changes in its epigenetic and spatial organization during spermatogenesis. Accurate control of pericentric heterochromatin is required for meiotic cell divisions and production of fertile and epigenetically intact spermatozoa. In this study, we demonstrate that pericentric heterochromatin is expressed during mouse spermatogenesis to produce major satellite repeat (MSR) transcripts. We show that the endonuclease DICER localizes to the pericentric heterochromatin in the testis. Furthermore, DICER forms complexes with MSR transcripts, and their processing into small RNAs is compromised in *Dicer1* knockout mice leading to an elevated level of MSR transcripts in meiotic cells. We also show that defective MSR forward transcript processing in *Dicer1* cKO germ cells is accompanied with reduced recruitment of SUV39H2 and H3K9me3 to the pericentric heterochromatin and meiotic chromosome missegregation. Altogether, our results indicate that the physiological role of DICER in maintenance of male fertility extends to the regulation of pericentric heterochromatin through direct targeting of MSR transcripts.

## INTRODUCTION

Spermatogenesis is a complex differentiation process including mitotic proliferation of spermatogonia, meiotic divisions of spermatocytes, and finally, morphological transformation of haploid round spermatids to mature spermatozoa. Post-transcriptional gene regulation during spermatogenesis is challenged by exceptionally broad expression of the genome in meiotic and early postmeiotic cells, and subsequent silencing of transcriptional activity due to chromatin compaction during late spermatogenesis (1). Late spermatocytes and round spermatids have unusually diverse transcriptomes, and in addition to numerous protein-coding mRNAs and their isoforms, they produce a broad spectrum of non-coding RNAs and intergenic transcripts (2). The functional importance of many of these transcripts has remained unknown. However, it is clear that distinct RNA regulatory mechanisms are required to control their fate, and to ensure the production of fertile and epigenetically intact spermatozoa.

We have previously shown that the function of endoribonuclease DICER in germ cells is essential for normal spermatogenesis; male mice lacking DICER in postnatal male germ cells are infertile due to severe defects in haploid differentiation (3,4), but the exact molecular mechanisms underlying the phenotype are not known. DICER has a well-characterized role in the processing of microRNAs (miRNAs) and small interfering RNAs (siRNA) that are important for post-transcriptional gene regulation and spermatogenesis (5,6). Emerging evidence suggests that DICER also has several non-canonical functions beyond miRNA/siRNA biogenesis, for example in transcriptional gene silencing at the chromatin level, as well as in RNA degradation and maintenance of genomic integrity (7). Although DICER predominantly localizes to the cytoplasm, some of its non-canonical functions may require nuclear localization. Indeed, several reports have provided evidence for the nuclear functions and chromatin association of mammalian DICER (8–13). Collectively, these data suggest that functional DICER can localize both to nucleus and cytoplasm to regulate gene expression by either miRNA-dependent or -independent mechanisms.

Nuclear DICER is involved in the formation of heterochromatin in lower organisms, such as the fission yeast, plants and flies (14,15). In mammals, DICER function has been linked to the control of heterochromatin by studies showing dysregulation of centromeric silencing in mouse embryonic stem cells and chicken–human hybrid DT40 cell line where DICER was conditionally deleted (16–18). Constitutive heterochromatin is found mainly at centromeres, that are essential for chromosomal segregation (19,20). On both sides of the centromere core region is a distinct chromatin structure, pericentric heterochromatin, that in mouse mainly consists of non-coding tandem repetitions called major satellite repeats (MSR) (21,22). Pericentric heterochromatin is bound by heterochromatin protein 1 (HP1) and marked by silencing histone modifications, such as trimethylation of lysine residue 9 of histone H3 (H3K9me3) and trimethylation of lysine residue 20 of histone H4 (H4K20me3) (23). Pericentric heterochromatin is typically organized into distinct nuclear domains called chromocenters, and its dynamic organization is known to be a prerequisite for early development and cellular differentiation (23). In differentiating male germ cells, pericentric heterochromatin has an important function in controlling global genome organization and meiotic chromosome interactions (24–28). Right after meiosis, pericentric heterochromatin is organized into a single chromocenter, which is thought to facilitate chromatin condensation during late spermatogenesis (29,30).

Despite the presence of silencing epigenetic marks, pericentric heterochromatin is transcribed to produce MSR transcripts (20). In mouse, pericentric heterochromatin transcription is cell cycle-regulated and MSR transcripts transiently increase in the late G1/early S phase and persist through mitosis (31). Transcriptional activity of pericentric heterochromatin has been shown particularly during cellular stress, cellular differentiation and early embryonic development, and MSR transcripts are also abnormally accumulated in many cancers (32–37). The mechanisms involved in the regulation of MSR transcription have remained poorly characterized. However, current evidence supports the functional significance of MSR transcription for example in the epigenetic silencing of pericentric heterochromatin and chromocenter formation (34,35,38–41).

Given the role of DICER in regulation of heterochromatin in non-mammalian organisms, we wanted to investigate if MSR expression is affected in the absence of DICER in a complex mammalian physiological context: during mouse male germ cell differentiation, which is characterized by transcriptional diversity and active makeover of the epigenome. We show that MSR transcripts are expressed during mouse spermatogenesis, especially in meiotic pachytene spermatocytes. We also show that in the absence of DICER, MSR transcript levels are elevated due to their defective processing, therefore highlighting a critical role for DICER as a regulator of MSR expression in spermatocytes. Furthermore, lack of DICER results in problems in meiotic chromosome segregation, thus providing an intriguing connection between MSR expression and the spermiogenic defects of DICER null mice.

## MATERIALS AND METHODS

### Animals

Mice were housed and fed in the controlled environment at the Central Animal Laboratory of the University of Turku, Finland. Germ cell-specific conditional *Dicer1* knockout (*Dicer1* cKO) mice were generated as previously described using Neurogenin3 (Ngn3) promoter-driven Cre expression (3). *Dicer* (fx/wt) littermates without Cre expression were used as controls. The mice were of mixed genetic background (C57Bl/6J and SV129). For wild type (WT) studies, we used either C57Bl/6J or C57BL/6N mouse strain. Experimentation with all animals and animal husbandry were carried out according to Finnish laws. All animal treatment and experiments followed the guidelines of Ethics of Animal Experimentation at the University of Turku in accordance with the Guide for Care and Use of Laboratory Animals (National Academy of Science).

### Immunofluorescence

4% paraformaldehyde (PFA) fixed and paraffin-embedded testis sections were deparaffinized (Xylene 3 × 3 min, 100% EtOH 2 × 3 min, 96% EtOH 2 × 3 min, 70% EtOH 2 × 3 min and Milli-Q water 1 × 5 min) and antigens were retrieved in citrate buffer (10 mM sodium citrate, pH 6.0) in the pressure cooker for 20 min. Slides were quenched with 100 mM NH_4_Cl for 5 min, washed with phosphate-buffered saline (PBS) and permeabilized with 0.5% Triton X-100 PBS for 10 min. After blocking in the blocking solution (2% normal donkey serum, 2% BSA, 0.1% Triton X-100 in PBS) for 30–60 min, primary antibody (Table 1) incubations were performed in the blocking solution for overnight at 4°C. Slides were washed in PBS with 0.1% Tween-20 (PBST) 3 × 10 min and incubated with secondary antibodies (Table 1) in the blocking solution for 1 h at Room Temperature (RT). Slides were washed with PBST 3 × 10 min, stained with 4′,6-diamidine-2′-phenylindole dihydrochloride (DAPI, Sigma-Aldrich, 0.25 mg/ml) for 10 min at RT and mounted with ProLong Diamond Antifade Mountant (Invitrogen). The signals were visualized by laser scanning confocal microscopy (Zeiss LSM780).

**Table 1.** Antibodies used in the study

Antibodies	Company (Cat. No.)	Applications
DICER (rabbit)	Sigma (SAB4200087)	IF (1:100), WB (1:200)
DICER (rabbit)	Bethyl Laboratories, Inc. (A301-936A)	IP, ChIP
SUV39H2 (rabbit)	Abcam (EPR18495)	ChIP
SUV39H2 (rabbit)	Proteintech Group, Inc. (11338-1-AP)	IP, WB (1:500)
SETDB1 (rabbit)	Proteintech Group, Inc. (11231-1-AP)	IP, WB (1:500)
HP1β (mouse)	Millipore (MAB3448)	IF (1:25)
H3K9me3 (rabbit)	Millipore (07-442)	IF (1:100), ChIP
MILI/PIWIL2 (mouse)	Sigma (MABE363)	IF (1:300)
TDRD1 (rat)	R&D Systems (MAB6296)	IF (1:200)
DDX25 (goat)	Santa Cruz Biotechnology (sc-51271)	IF (1:100)
phospho-Histone H2A.X (Ser139) (mouse)	Millipore (05-636)	IF (1:500)
phospho RNA Polymerase II (S2) (rabbit)	Bethyl Laboratories, Inc. (A300-654A)	WB (1:500)
β-TUBULIN (rabbit)	Cell Signaling (2128S)	WB (1:1000)
GAPDH (mouse)	Hytest Ltd. (5G4-6C5)	WB (1:3000)
Cytochrome C (mouse)	BD Biosciences (556433)	IF (1:300)
DDX4 (rabbit)	Abcam (ab13840)	WB (1:500)
YY1 (rabbit)	Bethyl Laboratories, Inc. (A302-779A-M)	WB (1:500)
Histone H3 (rabbit)	Cell Signaling (4499S)	WB (1:300)
TRIM33 (rabbit)	Bethyl Laboratories, Inc. (A301-060A-M)	WB (1:500), IP, IF (1:50)
TRIM28 (rabbit)	Bethyl Laboratories, Inc. (A304-145A-M)	IP
TRIM28 (rabbit)	Bethyl Laboratories, Inc. (A300-274A-M)	WB (1:500), IF (1:100)
Negative control IgG (mouse)	Santa Cruz Biotechnology (sc-2025)	IP,ChIP
Negative control IgG (rabbit)	Neomarkers (NC100-P1)	IP, ChIP
Donkey anti-mouse IgG Alexa Fluor 488 conjugate	Invitrogen (A-21202)	IF (1:1000)
Donkey anti-mouse IgG Alexa Fluor 594 conjugate	Invitrogen (A-21203)	IF (1:1000)
Donkey anti-rabbit IgG, Alexa Fluor 488 conjugate	Invitrogen (A-21206)	IF (1:1000)
Donkey anti-rabbit IgG Alexa Fluor 594 conjugate	Invitrogen (A-21207)	IF (1:1000)
Donkey anti-rat IgG Alexa Fluor 488 conjugate	Invitrogen (A-21208)	IF (1:1000)
Anti-rabbit HRP-conjugated	Cell Signaling (7074S)	WB (1:1000)
Anti-rouse HRP-conjugated	Cell Signaling (7076S)	WB (1:1000)
Anti-rabbit light chain HRP-conjugated	Millipore (MAB201P)	WB (1:1000)

### Electron microscopy

Testis samples were fixed in 5% glutaraldehyde and treated with a potassium ferrocyanide-osmium fixative. The samples were embedded in epoxy resin (Glycidether 100, Merck), sectioned, post-stained with 5% uranyl acetate and 5% lead citrate, and visualized on a JEOL 1400 Plus (JEOL Ltd., Tokyo, Japan) transmission electron microscope.

### Semi-quantitative RT–PCR

Total RNA from wild-type and *Dicer1* cKO testes or spermatocyte and round spermatids enriched by centrifugal elutriation (see below) were prepared using TRIzol (Invitrogen). 1 μg of RNA was treated with Turbo-DNase (Invitrogen) to remove DNA contamination. cDNA synthesis was carried out with random hexamers and the M-MLV reverse transcriptase (DyNamo cDNA synthesis kit, Finnzymes) according to manufacturer's instructions. *L19* was used as a reference gene. For strand-specific expression analysis of MSRs, either forward or reverse-specific primers were used for cDNA synthesis and *U1* was used as a reference gene, as described earlier (35). Finally, 5% of the reaction product was used as a template for PCR amplification. PCR was carried out with the following conditions: initial denaturation, 96°C for 3 min, and subsequently for 26–27 cycles, 95°C for 30 s, 57°C for 20 s, 72°C for 1 min, followed by final elongation at 72°C for 10 min. PCR products were run into 3–3.5% agarose gel. ImageJ software was used for quantification. The signal intensity of 308 bp MSR transcripts were normalized by dividing the MSR-tr signal in each sample with the signal of the reference gene expression in the same sample. A selected sample was used as a calibrator (see Figure legends) and set as ‘1’, and the expression of MSR-tr in other samples were presented relative to the calibrator expression. All primers are listed in the Table 2.

**Table 2.** Primers for RT-PCR and ChIP-PCR

Name	Primer sequences	*T*_a_ (°C)
L19	Fw, 5′- GGACAGAGTCTTGATGATCTC -3′	57
	Rev, 5′- CTGAAGGTCAAAGGGAATGTG -3′	
Ppia	Fw, 5′- GCCATGGTCAACCCCACCGT -3′	57
	Rev, 5′- TGCAAACAGCTCGAAGGAGACG -3′	
Major satellite	Fw, 5′- GACGACTTGAAAAATGACGAAATC -3′	57
	Rev, 5′- CATATTCCAGGTCCTTCAGTGTGC -3′	
Minor satellite	Fw, 5′- CATGGAAAATGATAAAAACC -3′	57
	Rev, 5′- CATCTAATATGTTCTACAGTGTGG -3′	
Line1	Fw, 5′- TTTGGGACACAATGAAAGCA -3′	60
	Rev, 5′- CTGCCGTCTACTCCTCTTGG -3′	
SineB1	Fw, 5′- GTGGCGCACGCCTTTAATC -3′	60
	Rev, 5′- GACAGGGTTTCTCTGTGTAG -3′	
IAP	Fw, 5′- AGCAGGTGAAGCCACTG -3′	62
	Rev, 5′- CTTGCCACACTTAGAGC -3′	
Gapdh	Fw, 5′- AGTGCCAGCCTCGTCCCGTA -3′	57
	Rev, 5′- AGGCGCCCAATACGGCCAAA -3′	
rDNA	Fw, 5′- GTAGTCGCCGTGCCTACCAT -3′	60
	Rev, 5′- TTTTCGTCACTACCTCCCCG -3′	

IAP, intracisternal A particle. *T*_a_, annealing temperature.

### RNA in situ hybridization

DIG-labelled (5′) LNA probe for MSR forward transcript (5′-TCTTGCCATATTCCACGTCC-3′) (Supplementary Figure S1A) (35) was purchased from Exiqon. DIG labeled Scramble probe was used as a negative control (Exiqon). 4% PFA fixed cryosections from WT, CTRL and *Dicer1* cKO adult testes were post-fixed and permeabilized with freshly prepared fixing solution containing 4% PFA, 0.5% Triton X-100 and 2 mM vanadyl ribonucleoside complex (VRC, New England BioLabs) in PBS for 20 min on ice with slow agitation. Sections were incubated with proteinase K (1.5 μg/ml) in PBS containing 35 mM EDTA for 20 min at 37°C, followed by the inactivation of proteinase K with 0.2% glycine in PBS for 5 min at 37°C. Sections were again post-fixed in 4% PFA in PBST (0.1% Triton X-100) for 10 min on ice and washed with PBS. 200 ml of pre-acetylation mix was prepared by adding 2.6 ml triethanolamine in DEPC-treated sterilized MQ water (pH 8). Slides were equilibrated in pre-acetylation mix for 10 min before adding 500 μl of acetic anhydride per 100 ml of pre-acetylation mix in mild agitation for 10 min, followed by washing three times with PBS. RNase treatment (Riboshredder, 50 U/ml, Epicenter Biotechnologies) was carried out in Tris buffer (pH 7) for 90 min in a moist chamber at 37°C, followed by pre-hybridization for 1 hour in the pre-hybridization mix containing 50% formamide, 2× saline sodium citrate (SSC), 10 mM VRC and 2 mg/ml bovine serum albumin (BSA, New England BioLabs) at 37°C. Hybridization was carried out in a hybridization mix (50% formamide, 2× SSC, 10% dextran sulfate, 10 mM VRC, 0.1% Tween-20, 2 mg/ml BSA, 10 mM citric acid, 500 μg heparin) containing 0.035 μM heat-denaturated (85°C for 5 min) MSR forward LNA probe in the sealed humidified chamber at 60°C for overnight. After post-hybridization washes (once with 2× SSC for 10 min, twice with 2× SSC for 30 min and twice with 0.1× SSC for 10 min) at 66–68°C, the slides were blocked with 4% BSA in the reaction buffer (100 mM Tris–HCl, 150 mM HCl, pH 7.5) for 40 min at 37°C. Slides were then incubated with alkaline phosphatase (AP)-conjugated anti-Digoxigenin-AP antibody (1:750, 11093274910, Roche) in 1% BSA in the reaction buffer at 37°C for 60 min. For combined immunostaining, slides were incubated again with anti-Digoxigenin-AP (1:1000) in combination with other primary antibodies (Table 1) in 1% BSA in PBS overnight at 4°C. Slides were washed with the reaction buffer with 0.1% Tween, incubated with secondary antibodies (Table 1) for 30 min at RT, washed again and equilibrated with detection buffer (100 mM Tris–HCl, 100 mM NaCl, 10 mM MgCl_2_, pH 8) for the detection of the DIG-labelled probes with the HNPP Fluorescent Detection Set (11758888001, Roche) according to manufacturer instructions. Slides were stained with DAPI (0.25 mg/ml) and mounted with ProLong Diamond Antifade Mountant (Invitrogen). Images were taken by laser scanning confocal microscopy (Zeiss LSM780). The MSR forward transcript signal was quantified by calculating average cytoplasmic intensity of CTRL and *Dicer1* cKO pachytene spermatocytes from three independent technical replicates (15–20 cells each) as shown in (Supplementary Figure S1G) using ImageJ.

### Preparation of testicular cell suspension and centrifugal elutriation

Adult testes were decapsulated and digested with 0.5 mg/ml Collagenase Type I (Worthington Biochemical Corporation) in 0.1% glucose PBS for 60–70 min at RT. Cell suspension was filtered through 100-μm filter and centrifuged at 1500 rpm for 5 min. Pellets were re-suspended in ice cold 0.1% glucose PBS and filtered again (40-μm filter). After final centrifugation, cell pellets were re-suspended in 10 ml of PBS. Pachytene spermatocytes and round spermatids were separated by using centrifugal elutriation (Beckman JE-6B Rotor Elutriator) at 2800 and 2000 rpm by adjusting different flow rates (42). The purity of each fraction was confirmed by microscopical examination of DAPI stained cells. Enriched spermatocytes and round spermatids were pelleted by centrifugation at 1500 rpm for 10 min at +4°C, snap-frozen in liquid nitrogen and stored at −80°C.

### Nuclear and cytoplasmic fractions

Germ cells from 18 days old mouse testes were released by Collagenase Type I as described above. Cells were mildly crosslinked in 0.1% PFA in PBS (pH 7.4) with slow rotation at RT for 10 min, followed by stopping the reaction with 125 mM glycine (Sigma) at RT for 5 min. Nuclear and cytoplasmic fractionation was done according to the protocol described earlier (43) with some modifications. Germ cells were lysed in hypotonic lysis buffer (40 mM Tris–HCl pH 7.4, 10 mM NaCl, 3 mM MgCl_2_, 0.005% NP-40) supplemented with fresh 1x Protease Inhibitor Cocktail (PIC, Roche) and 1 mM phenylmethanesulfonyl fluoride (PMSF) for 2 min in ice and centrifuged 2000 rpm for 2 min to collect supernatant as a cytoplasmic fraction. Remaining pelleted nuclei were washed twice with wash buffer I (50 mM Tris–HCl pH 7.4, 150 mM NaCl, 3 mM MgCl_2_, 0.25% NP-40, 0.25% Triton X-100, 1× PIC) and once with wash buffer II (1% Triton X-100, 1.2 mM EDTA, 167 mM NaCl, 16.7 mM Tris HCl pH 8.0, 1× PIC). The nuclear pellet was dissolved in high salt buffer (40 mM Tris–HCl pH 7.4, 400 mM NaCl, 1 mM EDTA, 3 mM MgCl_2_, 1 mM DTT, 1xPIC) for 15 min in ice and then diluted 5 times with dilution buffer (20 mM HEPES pH 7.4, 0.2 M NaCl, 0.5% Triton X-100, 5% glycerol, 1 mM EDTA, 1 mM EGTA, 3 mM MgCl_2_, 1xPIC) and centrifuged 10 000 rpm for 5 min to collect supernatant as a nuclear fraction. Samples were heated with Laemmli buffer at 90°C for 10 min to be used in western blotting as described below.

### Immunoprecipitation and western blotting

18 days mouse testes were decapsulated and lysed in a lysis buffer (50 mM Tris–HCl pH 7.5, 150 mM NaCl, 1.5 mM MgCl_2_, 1% Triton X-100, 0.1% NP-40, 1 mM PMSF, 1× PIC and 1× phosphatase inhibitors cocktail (PhosSTOP, Roche) by rotating slowly at +4°C for 50 min. Lysate was centrifuged at 10000 rpm for 10 min, and the supernatant was precleared with Dynabeads Protein G (Invitrogen) for 1 h at +4°C. Precleared lysates were incubated with 2–3 μg of antibodies (Table 1) overnight in rotation at +4°C, followed by 2 h incubation with beads pre-blocked with 5% BSA in PBS. After three washes with wash buffer I (50 mM Tris–HCl pH 7.5, 150 mM NaCl, 2 mM MgCl_2_, 0.2% Triton X-100, 0.2% NP-40) and wash buffer II (50 mM Tris–HCl pH 7.5, 150 mM NaCl, 2 mM MgCl_2_, 0.1% Triton X-100, 0.1% NP-40), protein complexes were eluted in the Laemmli buffer by heating at 90°C for 10 min. For western blotting, proteins were separated on a 4–20% polyacrylamide gel (Mini-PROTEAN, Bio-Rad) and transferred onto PVDF membrane overnight on ice at 60 V. Membranes were incubated with primary and secondary antibodies (Table 1) in 4% skimmed milk powder in TBST (0.05% tween) for 1 hour at RT. The signal was visualized by western lightning ECL Pro (NEL122001EA, PerkinElmer) reagent and ImageQuant LAS 4000 Biomolecular Imager (GE Healthcare).

### Chromatin fractionation

Germ cells were prepared from 18 dpp mouse testes as described above. Chromatin fractionation was done as described earlier (44) with some modifications. Cell pellets were resuspended in Buffer A (10 mM HEPES pH 7.6, 10 mM KCl, 1.5 mM MgCl_2_, 0.34 M sucrose, 10% glycerol, 1× PIC, 1 mM PMSF and 0.3 U/μl RNasin Plus RNase Inhibitor [Promega]) and incubated for 15 min on ice. Suspension was loaded in the top of 0.8 M sucrose and centrifuged 10 000 rpm for 15 min to collect top part as a cytoplasmic fraction and bottom pellet as a nuclear fraction. Nuclei were washed three times with Buffer A containing 3 mM EDTA and 0.3 mM EGTA. Chromatin was further fractionated by washing nuclei with nuclear Buffer B (40 mM Tris–HCl pH 7.4, 1 mM EDTA, 3 mM MgCl_2_, 1 mM DTT, 1xPIC, and 0.2 U/μl RNasin Plus RNase Inhibitor) with increasing salt concentrations (150 mM, 300 mM and 600 mM NaCl) at +4°C for 15 min. Subsequenctly, fractions were diluted 5 times with dilution buffer (20 mM HEPES pH 7.4, 0.2 M NaCl, 0.5% Triton X-100, 5% glycerol, 1 mM EDTA, 1 mM EGTA, 3 mM MgCl_2_, 1 mM DTT, 1xPIC and 0.15 U/μl RNasin Plus RNase Inhibitor), and centrifuged 10 000 rpm for 5 min to collect supernatant. The fractions were subjected for western blotting. Alternatively, small and long RNA were fractionated from each fraction by mirVana miRNA Isolation Kit (Invitrogen) and RNA dot blot was performed as described below.

### Mass spectrometry

DICER was immunoprecipitated from 17–18 dpp mouse testes as described above. Beads were washed with Tris buffer (pH 8.0) and digested with trypsin at the Turku Proteomics Facility according to the standard protocol. Digested peptides were dissolved in 0.1% formic acid and samples were submitted to LC–ESI-MS/MS analysis, on a nanoflow HPLC system (Easy-nLC1200, Thermo Fisher Scientific) coupled to the Q Exactive mass spectrometer (Thermo Fisher Scientific, Bremen, Germany) equipped with a nano-electrospray ionization source. MS data was acquired automatically by using Thermo Xcalibur 3.1 software (Thermo Fisher Scientific). An information dependent acquisition method consisted of an Orbitrap MS survey scan of mass range 300–2000 *m*/*z* followed by HCD fragmentation for 10 most intense peptide ions. Data files were searched for protein identification using Proteome Discoverer 2.2 software (Thermo Fisher Scientific) connected to an in-house server running the Mascot 2.6.1 software (Matrix Science). Data was searched against SwissProt database (version 2018_04).

### Chromatin immunoprecipitation (ChIP)

Germ cell suspension was prepared from 18 dpp testes as described above. Cells were cross-linked with 1% PFA for 20 min at RT, and cross-linking was stopped with 125 mM glycine for 5 min at RT. Cells were pelleted by centrifugation at 500 × *g* for 10 min. ChIP assay was performed according to (45) with slight modifications. Cross-linked germ cells were resuspended and incubated in a cytosol lysis buffer (5 mM PIPES pH 8.0, 85 mM KCl, 0.5% NP-40, 1 mM PMSF, 1× PIC) at 4°C for 5 min. Nuclei were pelleted and resuspended in a nuclei lysis buffer (50 mM Tris pH 8.0, 10 mM EDTA, 1% SDS, 1 mM PMSF, 1× PIC) and snap frozen twice in liquid nitrogen. Subsequently, lysates were diluted 5 times with the ChIP dilution buffer (1.1% Triton X-100, 0.01% SDS, 1.2 mM EDTA, 167 mM NaCl, 16.7 mM Tris–HCl pH 8.0, 1 mM PMSF and 1× PIC), sonicated with the BioRuptor sonicator (Diagenode) for 45 min on ice to make chromatin fragments between 500 and 700 bp. After centrifugation at 13 000 rpm for 10 min, supernatant was precleared with Dynabeads protein G for 90 min at +4°C. Lysates were incubated with (2–3 μg) ChIP antibodies (Table 1) overnight at cold room, followed by 2 h incubation with beads pre-blocked overnight with a solution containing 5% BSA, 0.6% cold fish gelatin, 0.2 μg/ml yeast tRNA, 1.5 μg/ml mouse Cot1 DNA, 0.05% Triton X-100, 1 mM PMSF and 1× PIC in PBS. ChIP complexes were washed 3 times with low salt wash buffer (150 mM NaCl, 0.1% SDS, 20 mM Tris–HCl pH 8, 2 mM EDTA, 1% Triton X-100), 3 times with high salt wash buffer (500 mM NaCl, 0.1% SDS, 1% Triton X-100, 20 mM Tris–HCl pH 8, 2 mM EDTA), 3 times with LiCl wash buffer (0.25 M LiCl, 1% NP-40, 10 mM Tris–HCl pH 8, 1 mM EDTA, 1% sodium deoxycholate) and 2 times with TE buffer (10 mM Tris–HCl pH 8, 1 mM EDTA). Protein–DNA complexes were eluted twice with freshly prepared elution buffer (1% SDS, 0.1 M NaHCO_3_). After adding NaCl to a final concentration of 300 mM, cross-links were reversed by incubating at 65°C overnight, followed by addition of 6 μl of 0.5 M EDTA (pH 8), 10 μl of 1 M Tris–HCl (pH 6.5) and 6 μl of 20 mg/ml proteinase K and further incubation for one hour at 42°C. DNA was isolated by TRIsure (Bioline) and isopropanol precipitation and PCR was performed with primers listed in Table 2.

### MSR transcript binding and processing assays

Maj9-2 plasmid containing mouse major satellite repeat DNA (46) was digested with MssI (PmeI) according to Thermo Scientific instructions. DIG-labelled MSR transcripts were generated *in vitro* from linearized Maj9-2 plasmid using SP6/T7 Transcription Kit (Roche) and DIG RNA Labeling Mix (Roche) at 37°C for 3 h. DIG-labelled Control transcripts were generated from a mixture of pSPT18- and pSPT19-neo-DNA (provided by the kit) cleaved with EcoRI. After DNase digestion, DIG-labeled RNAs were purified by acid-phenol chloroform (pH 4.5) extraction (Invitrogen) and isopropanol precipitation. MSR transcript binding and processing assays were performed by incubating 5 μg of DIG-labeled MSR and control transcripts with DICER complexes that were immunoprecipitated from 18 dpp mouse testes as described above in the binding buffer (20 mM Tris–HCl pH 7.4, 50 mM NaCl, 5 mM MgCl_2_ and 0.3% glycerol) at 35°C for 60 min. For binding assay, beads containing immunoprecipitation complexes were washed and bound RNAs were extracted (TRIsure, Bioline), and either directly applied on nylon membrane (Hybond-N+, Amersham Biosciences, Little Chalfont, UK) for RNA dot blotting or run into a 2.75% denaturing formaldehyde agarose gel in HT buffer (47) and capillary transferred overnight onto a nylon membrane in 10xSSC at +4°C. For processing assay, total RNA was isolated from whole reaction mixture, run into a denaturing 15% polyacrylamide-urea gel in TBE buffer and transferred using a semi-dry transfer system (Trans-Blot Turbo, Bio-Rad) in 0.5× TBE, 20V for 90 min at +4°C. RNA was cross-linked onto the nylon membrane by UVP CL-1000 Ultraviolet Cross linker (400 mJ for 40 s). Membranes were blocked with 4% BSA in Maleic acid buffer (0.1 M Maleic acid, 0.15 M NaCl, pH 7.5) at RT for 60 min. Subsequently, membranes were incubated with the alkaline phosphatase (AP)-conjugated anti-DIG-AP antibody (1:10 000 dilution, 11093274910, Roche) in Maleic acid buffer containing 1% BSA for 30 min, washed with 0.3% Tween 20 in Maleic acid buffer and equilibrated in DIG detection buffer (0.1 M Tris–HCl, 0.1 M NaCl, pH 9.5). DIG signal was visualized by incubating with 5–10 drops of a chemiluminescent alkaline phosphatase substrate (CSPD ready-to-use, Roche) for 20 min at RT followed by detection with ImageQuant LAS 4000 Biomolecular Imager (GE Healthcare).

### DICER-RNA immunoprecipitation

Germ cell suspension was prepared from 17–18 dpp testes as described above. Cells were cross-linked with 1% PFA for 20 min at RT, and cross-linking was stopped with 125 mM glycine for 5 min at RT. Cells were pelleted by centrifugation at 500 × *g* for 10 min. RNA immunoprecipitation assay was performed according to the protocol by Sun BK and Lee JT (protocol PROT28 at https://www.epigenesys.eu/en/) with some modifications. Pellets were resuspended in lysis buffer (50 mM Tris pH 8.0, 10 mM EDTA, 1% SDS, 1 mM PMSF, 1× PIC and 0.8 U/μl RNasin Plus RNase Inhibitor) on ice for 60 min. Lysates were diluted five times with dilution buffer (1.1% Triton X-100, 0.01% SDS, 1.2 mM EDTA, 167 mM NaCl, 16.7 mM Tris–HCl pH 8.0, 1 mM PMSF and 1× PIC) followed by mild sonication by BioRuptor sonicator (Diagenode) for 10 min in ice cold condition. After centrifugation at 13000 rpm for 10 min, the supernatant was subjected to immunoprecipitation (4 μg of Anti-DICER) using the same protocol as for the ChIP (see above). Washed protein-RNA complexes were eluted twice from the beads with freshly prepared elution buffer (1% SDS, 0.1 M NaHCO_3_, and 0.8 U/μl RNase Inhibitor). After adding NaCl to a final concentration of 300 mM, cross-links were reversed by incubating in 300 mM NaCl at 65°C for 2 hours, followed by addition of 6 μl of 0.5 M EDTA (pH 8), 10 μl of 1 M Tris–HCl (pH 6.5), 6 μl of 20 mg/ml proteinase K and 0.25 U/μl RNase Inhibitor and incubating for 45 min at 42°C. RNA was extracted by TRIsure (Bioline) and isopropanol precipitation and treated with Turbo-DNase (Invitrogen) to remove DNA contamination. cDNA synthesis was carried out with random hexamers and the M-MLV reverse transcriptase (Finnzymes) according to manufacturer's instructions. PCR was performed with primers listed in Table 2.

### Dot blotting and northern blotting with DIG-labelled probes

Total RNA was isolated by TRIzol (Invitrogen) and treated with Turbo-DNase (Invitrogen) to remove DNA contamination. For RNA dot blotting, RNA was applied onto nylon membrane (Hybond-N+, Amersham Biosciences, Little Chalfont, UK) and cross-linked by UVP CL-1000 Ultraviolet Cross linker (400 mJ for 40 s). For the detection of long RNAs by northern blotting, around 30 μg of total RNA was separated in 2% denaturing formaldehyde agarose gel prepared in HT buffer (47) and RNA was transferred onto nylon membrane by capillary method using 10× SSC at +4°C overnight. For the detection of small RNAs by northern blotting, small RNAs were fractionated from adult testes using mirVana miRNA Isolation Kit (Invitrogen), and around 40 μg of small RNA was separated in 18% polyacrylamide-urea gel in TBE buffer and transferred onto membrane by semi-dry transfer system (Trans-Blot Turbo, BioRad) using 0.5× TBE buffer (18 V, 3 h, +4°C). RNA was cross-linked onto the membrane by UV (400 mJ for 60 s). Pre-hybridization was performed in prehybridization mix containing 50% formamide, 2× SSC, 10 mM VRC, 2 mg/ml BSA and 1 μg/ml mouse Cot1-DNA (Invitrogen) at 37°C in the sealed plastic bag for 1 hour. Hybridization was done at 38°C for dot blots, at 59°C for long RNA northern blots and at 40°C for small RNA northern blots for overnight in a hybridization mix containing the prehybridization mix plus 0.1% Tween-20, 0.25% CHAPS, 10% dextran sulfate and 0.065 μM of DIG-labelled forward MSR transcript LNA probes denatured by heating at 85°C for 10 min. Post-hybridization washes were done at 62°C for long RNAs and at 42°C for small RNAs (once with 2× SSC for 10 min, twice with 2× SSC for 30 min and once with 0.1× SSC for 10 min). Membranes were blocked and incubated with anti-Digoxigenin-AP antibody, and DIG detection was performed as described above.

### Flow cytometry

Adult CTRL and *Dicer1* cKO (*n* = 3) testes were dissected and the tunica albuginea was removed in a 10-cm Petri dish on ice. The seminiferous tubules were gently pulled apart and 10 ml of PBS was added. To break up the testicular tissue architecture the solution was pipetted up and down a couple of times, and finally transferred to a 15-ml Falcon tube on ice. The tubules were allowed to settle on the bottom, the supernatant was removed, and 10 ml of PBS was added. This step was repeated and at the end the supernatant was removed. Collagenase I solution (2 ml per tube; 0,1 mg/ml [Worthington, USA, #LS004196] in DMEM/F12) was added and the sample was incubated 5+5 min at 37°C, with a short vortexing in between. To inactivate collagenase I, 10 ml of PBS was added, the sample was mixed, and the tubules were allowed to sediment on ice as before. Supernatant was removed and the wash was repeated. Trypsin (0.6 mg/ml in PBS; Worthington, USA, #LS003703) plus DNase I (8 μg/ml, Sigma Aldrich, #DN25) was added 2 ml per tube and incubated 5 min at 37°C. To inactivate trypsin, 10 ml of 10% FBS was added and the solution was pipetted up and down several times to homogenize the sample. The solution was then passed through a 100-μm cell strainer and pelleted (600 × *g*, 5 min, 4°C). The supernatant was removed, and the cells were resuspended in PBS and washed once. Epididymal cells from adult WT mice were analyzed as controls for the gating strategy (Supplementary Figure S6A,B). Epididymides were dissected and single cell suspensions were prepared from cauda epididymis by mechanical dissociation, and enzymatic digestion as described above. Single cells from testes and epididymis were resuspended in 0.2% BSA, 5 mg/ml RNase A in PBS and incubated for 15 min at 37°C. Hoechst 33342 solution (12.5 μg/ml, Thermo Fisher, #62249) was prepared according to manufacturer's instructions, added to the samples and incubated for 10 min at RT. Samples were run and data were acquired with LSRFortessa flow cytometer (Becton Dickinson) equipped with a high-throughput sampler (HTS) in 96-well plate format. A 405-nm laser was used for excitation of Hoechst 33342 and emission wavelengths were collected with a 450/50-nm band pass filter. Data were analyzed with the FlowJo software (FlowJo LLC).

### DNA *in situ* hybridization

Testicular cell suspension was prepared from two CTRL and two *Dicer1* cKO mice as described above, and pellets were incubated in 75 mM KCl solution for 20 min at 37°C. Cells were mixed with a fixing solution (1% PFA and 0.15% Triton X-100 in PBS, pH 9.2), spread on slides and incubated in a humidified chamber for overnight at RT. Slides were dried at RT, rinsed with 0.4% Photo-Flo (Kodak) and air-dried again. *In situ* hybridization was performed using probes detecting X and Y chromosomes as described in the Mouse Aneuploidy Kit FISH Protocol (FMAC-01, Creative Bioarray). Finally, slides were incubated with DAPI (0.25 mg/ml) for 10 min at RT and mounted with ProLong Diamond Antifade Mountant (Invitrogen). Slides were imaged with 3i spinning disk confocal microscopy. The Y and X chromosome signals were manually counted from at least 500 round spermatids per mouse. Round spermatids were recognized on the basis of their size and a typical heterochromatin pattern visualized by DAPI staining.

### Statistical analyses

All data are presented as mean values ± SEM. Statistical significances between groups were determined using two-tailed *t*-test. *P*-values < 0.05 were considered to be statistically significant.

## RESULTS

### Pericentric heterochromatin is transcribed during spermatogenesis

**Figure 1.**
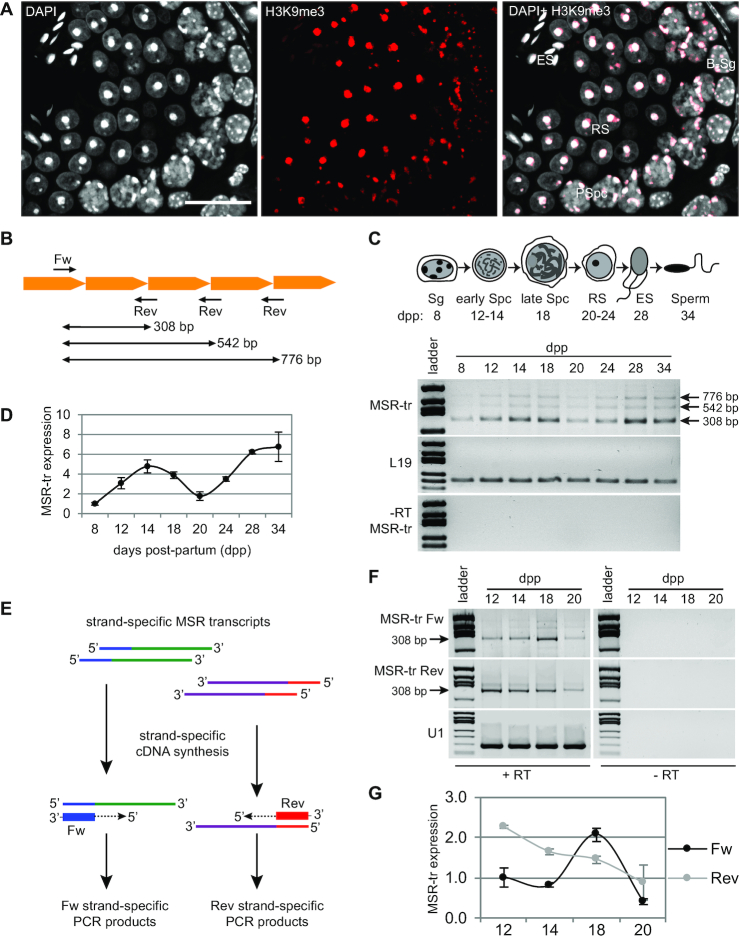
Major satellite repeats are transcribed during spermatogenesis. (**A**) Immunofluorescence on adult PFA-fixed paraffin-embedded testis sections (stage VI) using an antibody against H3K9me3 visualizes the accumulation of H3K9me3 signal at chromocenters of spermatogonia, spermatocytes and round spermatids. B-Sg, type B spermatogonium; PSpc, pachytene spermatocyte; RS, round spermatid; ES, elongating spermatid. Scale bar: 25 μm. (**B**) Schematic diagram showing the location of forward (Fw) and reverse (Rev) primers for the amplification of major satellite repeat (MSR) transcripts. Each pointed orange block corresponds to a consensus MSR sequence. See also Supplementary Figure S1A. (**C**) MSR transcripts (MSR-tr) are expressed during the first wave of spermatogenesis. Testis samples were collected from juvenile WT mice at different time points and MSR-tr expression was analyzed by semi-quantitative RT-PCR. *L19* was used as a reference gene. The reactions without a reverse transcriptase enzyme (-RT) control genomic DNA contamination. (**D**) The intensities of MSR-tr 308 bp bands as in (c) from two independent experiments were quantified using ImageJ and normalized to the intensity of *L19* band in the same sample. MSR-tr expression is shown relative to 8 days post-partum (dpp) sample that was set as ‘1’; *n* = 2, error bars represent standard errors of the mean (SEM). (**E**) Schematic diagram of strand-specific RT-PCR using either Fw or Rev primers in the reverse transcription reaction to specifically amplify only forward or reverse strand. (**F**) Expression of MSR forward (Fw) and reverse (Rev) strands in testis samples collected at different time points during the first wave of spermatogenesis. *U1* small nuclear RNA was used as a reference gene. (**G**) The intensities of MSR-tr 308 bp bands from two independent experiments were quantified using ImageJ and normalized to *U1* expression in the same sample. Expression is shown relative to MSR Fw 12 dpp sample that was set as ‘1’; *n* = 2, SEM.

Male germ cells are known to transcribe their genome widely, but the transcriptional activity of pericentric heterochromatin has remained elusive. First, we wanted to visualize pericentric heterochromatin in male germ cells to get a better view on the dynamic changes in its organization during the progress of spermatogenesis in mice. To this end, we performed immunofluorescence on adult testes using an antibody against H3K9me3 (Figure 1A), a histone modification that has been shown to be enriched at the pericentric heterochromatin (48–50). We found that pericentric heterochromatin foci were enriched with H3K9me3 already in spermatogonia, and maintained in spermatocytes. After meiotic divisions, pericentric heterochromatin was organized into a single H3K9me3-positive chromocenter in round spermatids (Figure 1A). H3K9me3 signal finally disappeared in condensing elongating spermatids, which is in line with the replacement of the majority of histones by sperm-specific chromatin proteins, protamines, in these cells (51).

We then studied the expression dynamics of pericentric heterochromatin-derived major satellite repeat (MSR) transcripts during spermatogenesis by semi-quantitative RT-PCR using primers that give different-sized products depending on how many sequential repeats they amplify (Figure 1B, Supplementary Figure S1A). As samples, we used juvenile testes that were collected at the specific time points that correspond to the appearance of distinct types of differentiating germ cells during the first wave of spermatogenesis (52) (Figure 1C). Testes collected at 8 days post-partum (dpp) contain Sertoli cells and proliferating spermatogonia. At 12 dpp, the most advanced germ cells have already entered meiosis, and the time points 14 dpp and 18 dpp correspond to the appearance of mid-pachytene and late-pachytene/diplotene phase spermatocytes at prophase I of meiosis, respectively. At 20 dpp, the first round spermatids have appeared, and at 28 dpp the elongation of spermatids has begun. We detected abundant amplification of MSR transcripts in all studied time points. Interestingly, the expression level was not steady during the progress of spermatogenesis, but peaked at time points 14–18 dpp corresponding to the appearance of pachytene spermatocytes (Figure 1C, D). This finding suggests that meiotic pachytene spermatocytes express a high level of MSR transcripts.

Transcription of MSR sequences can occur from both strands (53), and it has been shown that forward and reverse strands are differentially expressed during early mouse development (35). To clarify the expression of MSR sequences from each strand, we performed reverse transcription using strand-specific primers followed by PCR (Figure 1E). Both forward and reverse strands were shown to be expressed, but interestingly, their expression patterns during the first wave of spermatogenesis varied and only forward strand expression peaked at 18 dpp (Figure 1F, G).

### MSR expression is induced in *Dicer1* knockout germ cells

We have previously generated a mouse model with a germ cell-specific deletion of *Dicer1* in early postnatal spermatogonia using *Neurogenin 3* (*Ngn3*) promoter-driven expression of Cre recombinase (*Dicer1* cKO) (3,4). *Dicer1* cKO male mice are infertile due to defective postmeiotic male germ cell differentiation. In the initial analysis of *Dicer1* cKO mice, we observed that the expression of MSR transcripts was induced in the adult testis (3). To study this intriguing finding in more detail, MSR expression was followed during the first wave of spermatogenesis in *Dicer1* cKO mice. As controls, we used WT-like littermates that were of mixed genetic background in contrast to the inbred WT mice used in Figure 1. On a mixed genetic background, the abundance of MSR transcripts followed a similar pattern during the first wave of spermatogenesis as shown above for WT testis (compare Figure 1C,D and Supplementary Figure S1B,C). Interestingly, the relative level of MSR expression was generally higher in *Dicer1* cKO testes than in control testes (Supplementary Figure S1B,C). Despite prominently induced expression at 18 dpp in cKO testes, MSR transcript levels were downregulated at later time points (20 dpp), as also seen in the control testes (Supplementary Figure S1B,C).

**Figure 2.**
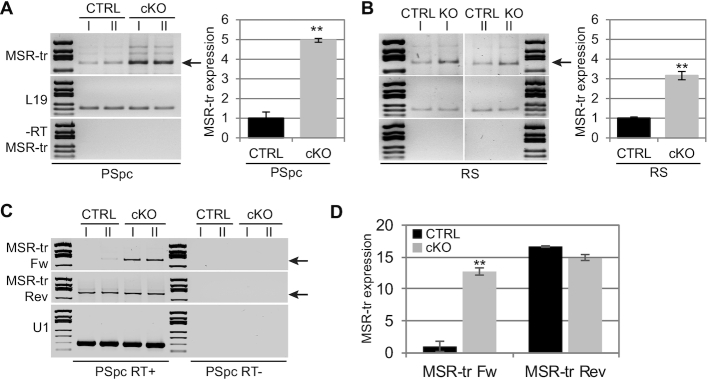
Major satellite repeat expression is misregulated in *Dicer1* knockout germ cells. (A, B) Semi-quantitative RT-PCR for enriched populations of pachytene spermatocytes (PSpc) (**A**) and round spermatids (RS) (**B**) from control and *Dicer1* cKO mice revealed increased levels of MSR transcripts in both cell types, particularly in spermatocytes, in the absence of DICER. MSR-tr 308 bp band (arrow in A) intensities were quantified using ImageJ and normalized to L19 band intensity in each sample. MSR-tr expression is shown relative to the CTRL samples that were set as ‘1’; *n* = 2, SEM, ** *P*< 0.01. (**C**) Strand-specific expression of MSR was analyzed in control and *Dicer1* cKO spermatocytes. Only forward strand of MSR was found more highly expressed in *Dicer1* knockout spermatocytes. (**D**) 308 bp MSR-tr signal (arrows in C) was quantified and normalized to *U1* signal in each sample. MSR-tr Fw and Rev expression in cKO is shown relative to the CTRL Fw sample that was set as ‘1’; *n* = 2, SEM, ** *P*< 0.01. See also Supplementary Figure S1B–F.

Elevated expression of MSR transcripts at 18 dpp cKO testes suggests that the defect in the regulation of MSR transcript levels takes place in pachytene spermatocytes that are the most abundant in testis at this time point. To confirm this, we enriched pachytene spermatocytes and round spermatids from control and cKO adult mouse testes by centrifugal elutriation and performed semi-quantitative RT-PCR. The cell fractions were calculated to be >85% pure as evaluated by microscopical analysis of DAPI-stained cells (Supplementary Figure S1D). As expected, MSR transcripts were readily detected in spermatocytes and round spermatids (Figure 2A, B). Importantly, we detected a significantly higher expression of MSR in *Dicer1* cKO germ cells, especially in pachytene spermatocytes. Next, we used strand-specific RT-PCR to study if the absence of DICER affects the expression of MSR in a strand-specific manner. Interestingly, only expression of the forward strand was higher in *Dicer1* cKO pachytene spermatocytes, while expression of the reverse strand was not affected (Figure 2C, D). Furthermore, higher expression of the forward strand was also confirmed in *Dicer1* cKO testes during the first wave of spermatogenesis (Supplementary Figure S1E, F).

### Processing of MSR transcripts is compromised in the absence of DICER

**Figure 3.**
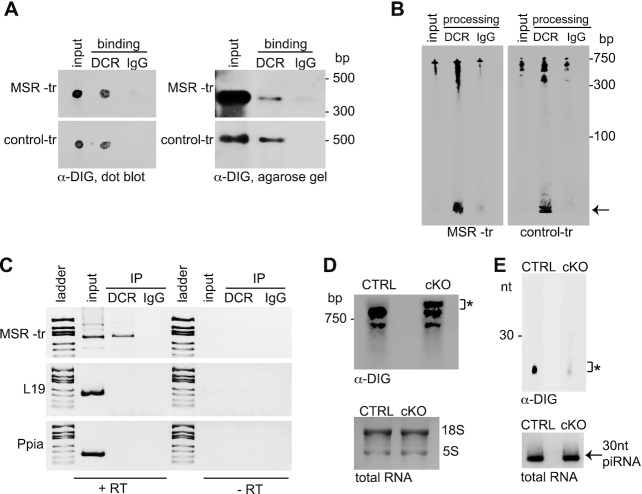
DICER binds and processes major satellite repeat transcripts. (**A**) DICER interacts with MSR transcripts (MSR-tr) *in vitro*. DIG-labelled *in vitro* transcribed MSR or randomly-selected control transcripts were incubated with DICER-complexes (DCR) immunoprecipitated from mouse testes. Rabbit IgG (IgG) was used as a negative control for immunoprecipitation. Binding was detected by anti-DIG antibody both by dot blotting (left panel) or by running bound RNA into an agarose gel followed by transfer to a membrane (right panel). Both MSR-tr and control-tr bound to DICER complexes, while no binding was detected in the negative IgG control. (**B**) DICER complexes are able to process MSR transcripts *in vitro*. DIG-labeled MSR or control transcripts were incubated with testis DICER-complexes and RNA was run into a polyacrylamide gel for anti-DIG detection. Both MSR and control transcripts were processed into smaller products by DICER complexes (black arrow) but not by control IgG immunoprecipitations. (**C**) MSR transcripts form complexes with DICER in the testis *in vivo*. Cross-linked testicular extracts were immunoprecipitated by anti-DICER or control rabbit IgG antibodies, and co-precipitated RNAs were detected by RT-PCR using primers specific for MSR transcripts. *L19* and *Ppia* (peptidylprolyl isomerase A) primers were used as negative controls. In the -RT reaction, cDNA synthesis reaction was performed in the absence of reverse transcriptase. (**D**) Long MSR transcripts accumulate in *Dicer1* cKO testes. Total RNA was extracted from control (CTRL) and *Dicer1* cKO (cKO) testes and run into an agarose gel for Northern blotting using DIG-labeled MSR forward transcript probes. The longer MSR transcripts that appear only in cKO testes are indicated by an asterisk. Total RNA was stained to visualize 18S and 5S rRNAs for the validation of equal loading of RNA. (**E**) MSR transcript-derived small RNAs are dramatically reduced in *Dicer1* cKO testes. Small RNAs from control and *Dicer1* cKO testes were run into a polyacrylamide gel and MSR forward transcript-derived small RNAs (asterisk) were detected by Northern blotting. Small RNAs were stained to visualize ∼30 nt piRNAs (black arrow) for the validation of equal loading of RNA. All panels show a representative figure of an experiment that was independently repeated at least two times.

The well-known function of DICER in RNA processing prompted us to investigate the possibility if upregulation of MSR transcript expression could originate from defective DICER-mediated post-transcriptional processing of MSR transcripts. We first verified that DICER that was immunoprecipitated from 17 to 18 days mouse testes was able to bind to *in vitro*-transcribed MSR transcripts and process them into small RNAs (Figure 3A, B). Notably, the control transcript (*Neomycin* mRNA) was also similarly bound and processed by testicular DICER complex, suggesting a broad substrate specificity. Direct processing of MSR transcripts *in vivo* requires that DICER and MSR transcripts associate with each other, and therefore, we analyzed the presence of MSR transcripts in DICER-complexes that were immunoprecipitated from mouse testes. Importantly, MSR transcripts, but not control transcripts (*L19* and *Ppia*), were readily detected in DICER complexes, confirming a specific interaction of DICER with MSR transcripts *in vivo* (Figure 3C).

To study if there are any changes in the size distribution of MSR transcripts in the testis in the absence of DICER, total RNA was extracted from control and *Dicer1* cKO testes and ran onto an agarose gel for detection of forward MSR transcripts using a DIG-labeled probe. Interestingly, while MSR transcript products sized ∼300–800 nucleotides were detected in both control and *Dicer1* cKO testes, an additional longer product, that was not detected in control testes, accumulated in cKO testes (Figure 3D). The existence of this longer MSR transcript exclusively in knockout testes may indicate defective processing of MSR transcripts in *Dicer1* cKO mice. Indeed, Northern blotting of small RNAs isolated from control and *Dicer1* cKO testes showed a dramatic decrease in the amount of forward MSR transcript-derived small RNAs (Figure 3E), indicating that the elevated levels of MSR transcripts in *Dicer1* cKO testis are due to defective DICER-mediated processing of MSR transcripts.

### DICER localizes to the chromatin with MSR forward transcripts

**Figure 4.**
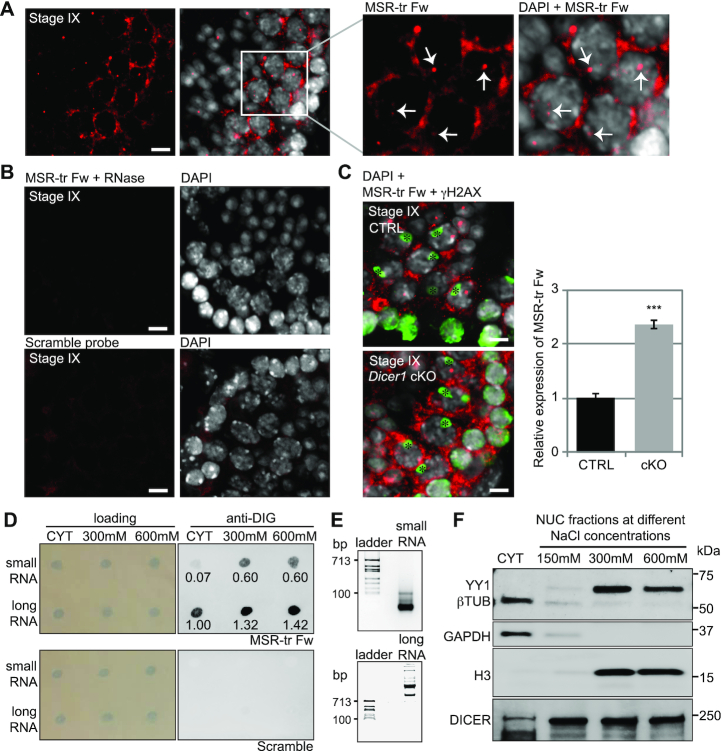
DICER and major satellite repeat transcripts associate with chromatin. (**A**) *In situ* hybridization using a LNA probe for the forward strand-derived MSR transcripts (MSR-tr Fw, red) on WT testis cryosections. Strong signal was detected in the cytoplasm of late pachytene spermatocytes at stage IX of the seminiferous epithelial cycle. MSR-tr signal was also detected in the nucleus (arrows). Nuclei were stained by DAPI (grey). (**B**) The forward MSR transcript signal disappeared when the sections were treated with RNase prior to hybridization, demonstrating that the signal originated from RNA transcripts. No signal was detected using a scramble probe. Scale bars for A and B: 10 μm. (**C**) Relatively stronger expression of the forward MSR transcripts (red) was detected by *in situ* hybridization in *Dicer1* cKO spermatocytes compared to the control. To facilitate the recognition of pachytene spermatocytes, the sections were immunostained using an antibody against phosphorylated gamma-H2AX (γH2AX, green), which detects double-strand breaks and labels the sex body (asterisks) in pachytene spermatocytes. Scale bar: 10 μm. The graph shows quantification of average signal intensity of cytoplasmic MSR Fw transcripts signal in CTRL and *Dicer1* cKO pachytene spermatocytes. The signal was quantified from three independent technical replicates (15–20 cells each); SEM, ***P< 0.001. See also Supplementary Figure S1G. (**D**) Chromatin fractionation was performed from nuclear extracts of 18 dpp mouse testicular cells using increasing concentrations of NaCl, and RNA was extracted from the cytoplasmic fraction as well as from 300 mM and 600 mM NaCl chromatin fractions. MSR transcripts were detected separately from long (>200 nt) or short (<200 nt) RNA fraction with the LNA probe against the forward MSR transcript. Long MSR transcripts were found both in the cytoplasmic and chromatin fractions, while MSR transcript-derived small RNAs were mainly detected in the chromatin fractions. The numbers below the anti-DIG signal indicate relative intensities of MSR-tr Fw signal quantified by ImageJ. The signal intensities are shown relative to the cytoplasmic long RNA sample that was set as ‘1’. (**E**) Separation of total RNA into long and small RNA fractions was validated by agarose gel electrophoresis followed by ethidium bromide staining. (**F**) Immunoblotting of cytoplasmic and chromatin fractions with anti-DICER antibody demonstrated the presence of DICER in all fractions. Cellular fractions were validated by immunoblotting with antibodies against cytoplasmic proteins (β-TUBULIN, GAPDH) and chromatin-associated proteins (YY1, H3). All panels show a representative figure of an experiment that was independently repeated at least two times.

Next we wanted to explore the subcellular localization of DICER and MSR transcripts in more detail to be able to better understand the function of DICER in the control of MSR expression. Because only forward MSR transcripts were upregulated in *Dicer1* cKO germ cells, we performed *in situ* hybridization on adult testis cryosections using a probe specifically detecting the forward transcript. Signal was prominent in the cytoplasm of late pachytene spermatocytes (Figure 4A). Although most of the signal was cytoplasmic, clear nuclear foci were also detected (Figure 4A). A scramble LNA probe was used as a negative control, and the specificity of the signal was also validated by RNase treatment prior to hybridization with the forward MSR probe (Figure 4B). Relatively stronger MSR forward transcript signal was detected in the cytoplasm of *Dicer1* cKO late pachytene spermatocytes when compared to the control (Figure 4C, Supplementary Figure S1G), which is in line with the increased expression of MSR forward strand in *Dicer1* cKO spermatocytes as detected by RT-PCR (Figure 2C, D).

To provide an additional piece of evidence for the presence of MSR forward transcripts in the nuclear compartment, we performed chromatin fractionation from 18 dpp testicular cells using increasing concentrations of NaCl. Chromatin-associated proteins and RNAs become more soluble with increasing concentration of NaCl, and molecules that are strongly bound to DNA are expected to elute with high salt, whereas loosely bound ones will elute with low salt (44). We separated long (>200 nt) and small RNAs (<200 nt) from the cytoplasmic and chromatin fractions and performed dot blotting with DIG-labeled MSR forward probe. Long MSR forward transcripts were detected both in cytoplasmic and chromatin fractions (Figure 4D). In contrast, small RNAs derived from MSR forward transcripts were readily detected in the chromatin fraction, but only very weakly in the cytoplasmic fraction (Figure 4D), suggesting that they mainly localize to the chromatin. RNA fractionation into the pools of long and small RNAs before dot blotting was validated by gel electrophoresis (Figure 4E). Interestingly, immunoblotting of the samples from same subcellular fractions revealed that DICER was also present in the chromatin fractions in addition to its well-known cytoplasmic localization (Figure 4F). DICER signal was still detected together with nucleosomal histone H3 in fractions eluted with the highest NaCl concentrations (600 mM), suggesting a tight association with chromatin. These experiments showed that DICER associates with chromatin together with MSR forward transcripts in male germ cells.

To get a better overall view on the localization pattern of DICER during spermatogenesis, we performed immunofluorescence analysis. As expected, the majority of DICER signal was found in the cytoplasm of differentiating male germ cells and its expression peaked in pachytene spermatocytes, i.e. the same cell type where the forward MSR transcripts were also expressed at the highest level (Supplementary Figure S2). The cytoplasmic DICER in pachytene spermatocytes was found to co-localize to cytoplasmic granular structures with PIWIL2/MILI (Piwi-like protein 2), TDRD1 (Tudor domain-containing protein 1), and Cytochrome C (Supplementary Figure S3). These proteins are known to localize to a germ cell-specific ribonucleoprotein granule, intermitochondrial cement (IMC), a structure which has been associated with post-transcriptional RNA processing, particularly processing of PIWI-interacting RNAs (piRNAs) (1). Thus, cytoplasmic DICER appears to be associated with the IMC in pachytene spermatocytes. Despite the predominant localization of DICER in the IMC, the morphology of IMC or chromatoid body (CB), a closely related germ granule in round spermatids (1), was not affected in *Dicer1* cKO mice, as judged by electron microscopy analysis (Supplementary Figure S4A). Furthermore, localization of the IMC components PIWIL2 and TDRD1 and the CB component DDX25 was not affected in *Dicer1* cKO germ cells (Supplementary Figure S4B).

**Figure 5.**
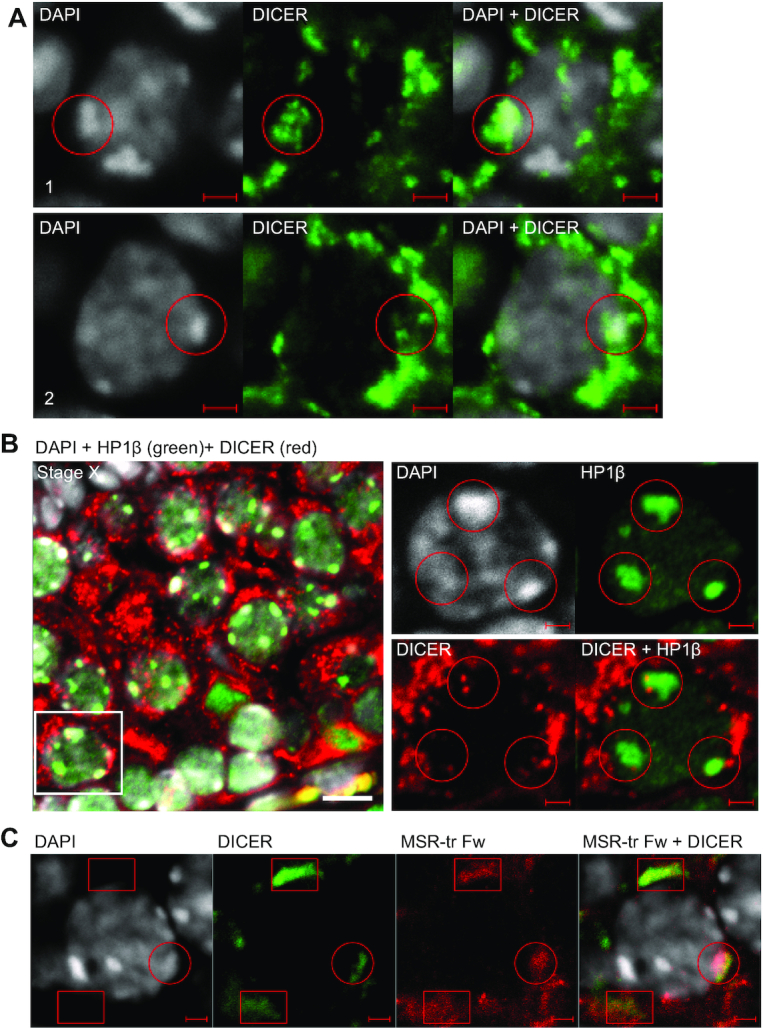
DICER localization in spermatocytes is associated with heterochromatin areas and MSR forward transcripts. (**A**) A panel shows a selected late pachytene spermatocyte from a PFA-fixed paraffin-embedded WT adult mouse testis section (stage IX) that was immunostained using an anti-DICER antibody (green). Nuclei were stained with DAPI (gray). Two different layers of the confocal Z-stack of the same cell are shown (1 and 2). DICER-positive foci are found in the nucleus, and the signal overlaps with DAPI-bright heterochromatin areas. Two different areas inside the same nucleus are indicated with red circles. Scale bars: 2 μm. (**B**) Nuclear DICER (red) is associated with heterochromatin as shown by co-immunostaining with anti-HP1β (green) on paraffin-embedded testis sections (stage X). Scale bar: 10 μm. Higher magnification images in the right panel show a single layer from a confocal stack to visualize an example spermatocyte indicated with a white box in the left panel. HP1β-positive heterochromatin areas are associated with DICER-positive foci (red circles). Scale bars in the high magnification images: 2 μm. (**C**) Immunofluorescence staining using an anti-DICER antibody (green) was combined with *in situ* hybridization using the MSR-tr Fw probe (red) on WT adult mouse testis cryosections. DICER partially co-localizes with MSR forward transcripts in the cytoplasm (red boxes) and in the nucleus (a red circle) of spermatocytes. Scale bars: 2 μm. All panels show a representative figure of an experiment that was independently repeated at least two times. See also Supplementary Figures S2–S4 and Supplementary Videos S1 and S2.

In addition to the prominent cytoplasmic localization, we indeed detected DICER-positive foci in germ cell nuclei, particularly in late pachytene spermatocytes at stages VII to X of the seminiferous epithelial cycle (Figure 5A, Supplementary Figure S2). Cell fractionation also confirmed the presence of DICER in the nuclear fraction (Supplementary Figure S2). Nuclear DICER foci appeared to localize in the close vicinity of DAPI-bright (Figure 5A, Supplementary Video S1) and HP1β-positive (Figure 5B, Supplementary Video S2) heterochromatin areas. Importantly, *in situ* hybridization using a probe recognizing forward MSR transcript combined with immunofluorescence using an anti-DICER antibody demonstrated that both nuclear and cytoplasmic DICER partially overlapped with MSR transcript signal (Figure 5C). These data combined with the immunoprecipitation data (Figure 3C) indicate that DICER associates with forward MSR transcripts and heterochromatin nuclear domains in spermatocytes.

### DICER directly interacts with pericentric heterochromatin in spermatocytes

**Figure 6.**
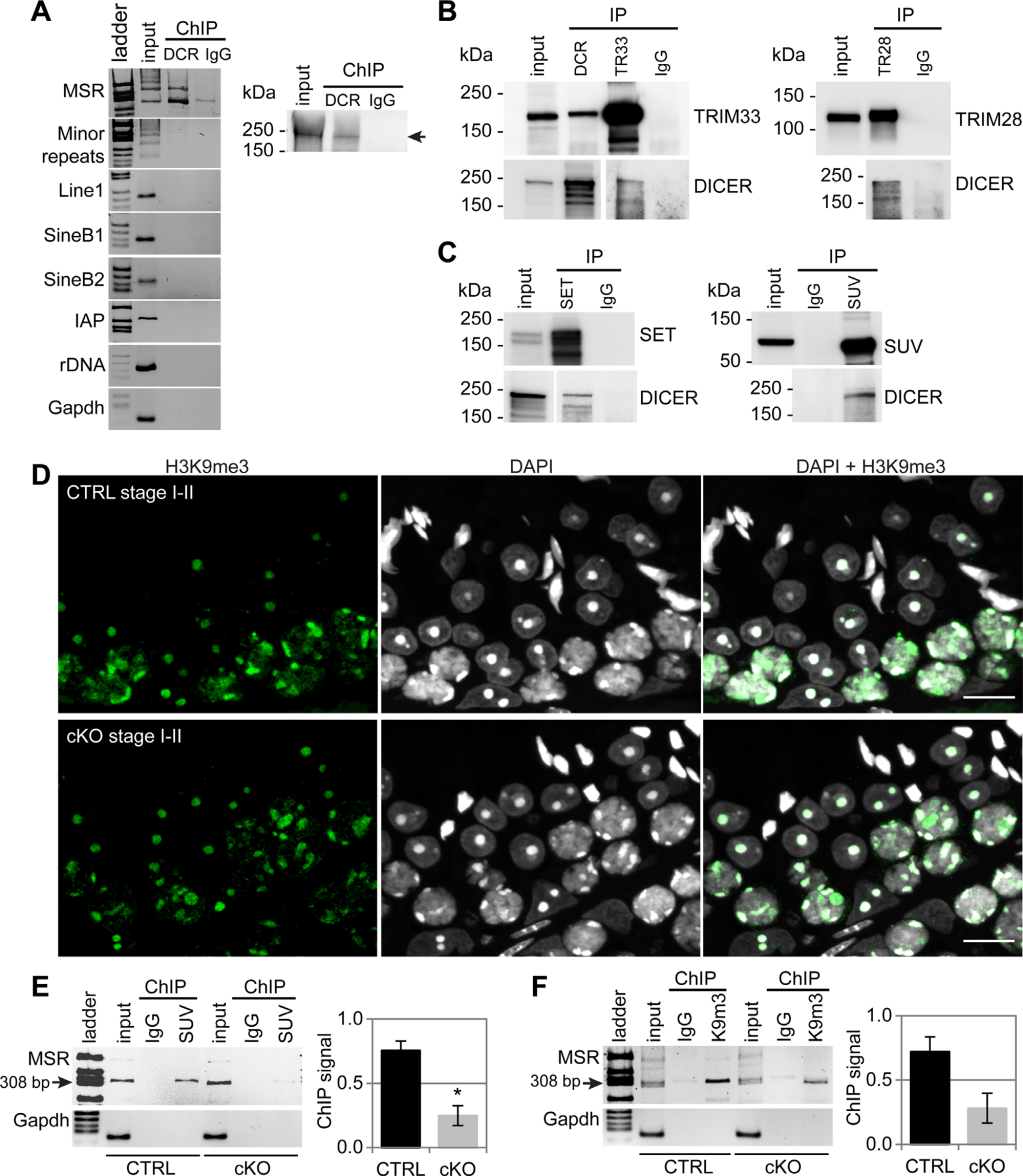
Epigenetic status of the MSR chromatin is disrupted in *Dicer1* knockout testes. (**A**) Chromatin immunoprecipitation (ChIP) with an anti-DICER (DCR) antibody followed by PCR using primers specific for different repeat sequences revealed the association of DICER with the MSR-containing genomic regions. DICER did not associate with minor satellite repeats, various transposon sequences (Line1, SineB1, SineB2, IAP) or ribosomal DNA repeats (rDNA). Rabbit IgG (IgG) was used as a negative control for immunoprecipitation. The primers for *Gapdh* gene were used as a negative control for PCR. The top right panel shows immunoblotting with an anti-DICER antibody to validate the performance of the antibody in the ChIP assay. (**B**) Immunoprecipitation using antibodies against DICER, TRIM33 and TRIM28 followed by western blotting with the same antibodies validated the interaction of DICER (DCR) with TRIM33 (TR33) and TRIM28 (TR28). LYS, lysate; IP, immunoprecipitation; IgG, control IP with rabbit IgG. (**C**) DICER interacts with histone methyltransferases in the testis. Immunoprecipitation using antibodies against SETDB1 (SET) and SUV39H2 (SUV) followed by immunoblotting with SETDB1, SUV39H2 and DICER antibodies. LYS, lysate; IP, immunoprecipitation; IgG, control IP with rabbit IgG. (**D**) Localization of H3K9me3 appears unaffected in *Dicer1* cKO germ cells. PFA-fixed paraffin-embedded controls (CTRL) and *Dicer1* cKO testis sections were immunostained with an anti-H3K9me3 antibody. Scale bar: 10 μm. Panels A-C show a representative figure of an experiment that was independently repeated at least two times. (**E**) ChIP with anti-SUV39H2 revealed that the association of SUV39H2 with MSRs is reduced in *Dicer1* cKO adult testis. (**F**) Overall H3K9me3 (K9m3) signal on MSR genomic regions appeared reduced in *Dicer1* cKO adult testis. The graphs in panels E and F show the quantification of the ChIP-PCR signal (308 bp band) in two independent experiments. The ChIP signal was normalized to the input signal. Signal intensities are shown relative to the CTRL ChIP samples that were set as ‘1’; *n* = 2, SEM, *P< 0.05. See also Supplementary Figure S5 and Supplementary Table S1.

The presence of DICER in the chromatin fraction prompted us to further explore whether DICER could be present in the pericentric heterochromatin to directly control MSR regions. To this end, we did chromatin immunoprecipitation using an anti-DICER antibody followed by PCR (ChIP-PCR) using MSR-specific primers. These experiments indeed confirmed the interaction of DICER with the genomic sequence of MSR in the pericentric heterochromatin (Figure 6A). In contrast, DICER did not associate with other repeat sequences, such as minor satellite repeats and transposons LINE1, SINEB1 and SINEB2 (Figure 6A), corroborating our earlier RT-PCR results that did not show any significant differences in the expression of these transcripts in *Dicer1* cKO when compared to the control testis (3).

To understand how DICER could be recruited to the pericentric heterochromatin, we studied the association of DICER with heterochromatin regulators. First, we performed mass spectrometric analysis of DICER-interacting proteins in 17–18 dpp mouse testes, and interestingly, we identified several potential nuclear interaction partners for DICER (Supplementary Table S1). These included KHDRBS1/SAM68 (KH domain-containing, RNA-binding, signal transduction-associated protein 1), snRNP200 (U5 small nuclear ribonucleoprotein 200 kDa helicase), hnRNP-M (Heterogeneous nuclear ribonucleoprotein M), PSPC1 (Paraspeckle component 1), SFPQ (Splicing factor, proline- and glutamine-rich), TRIM28 (Tripartite motif-containing 28) and TRIM33 (Tripartite motif-containing 33). Interestingly, TRIM28 and TRIM33 have been shown to act as scaffolding proteins that can recruit a variety of epigenetic modifiers to chromatin, including histone methyltransferases that are responsible for trimethylation of histone H3 at lysine 9 (54–60). The interactions of DICER with TRIM28 and TRIM33 were validated by immunoprecipitation followed by western blotting (Figure 6B). Immunofluorescence analysis confirmed the nuclear localization of TRIM28 and TRIM33 and their expression in the same cell types as DICER during spermatogenesis (Supplementary Figure S5A). Furthermore, co-immunoprecipitation experiments revealed that DICER can be found in complexes with H3K9 methyltransferases SUV39H2 (Suppressor of variegation 3–9 homolog 2) and SETDB1 in the mouse testis (Figure 6C). Altogether, these results suggest that the association of DICER with pericentric heterochromatin is mediated by the interaction with heterochromatin regulators.

We next studied if the defects in DICER-mediated MSR transcript processing have any consequences on the epigenetic status of pericentric heterochromatin. H3K9me3 or heterochromatin protein HP1β localization patterns did not show any obvious changes in adult *Dicer1* cKO testes (Figure 6D, Supplementary Figure S5B,C). However, ChIP-PCR revealed a reduced association of SUV39H2 and H3K9me3 with MSR chromatin in adult *Dicer1* cKO testes (Figure 6E,F). Due to variation between the biological replicates, this reduction reached a statistical significance only for SUV39H2. In conclusion, lack of DICER-dependent activities appears to imbalance the epigenetic status of pericentric heterochromatin by reducing the recruitment of SUV39H2 and the level of H3K9me3.

### Deletion of *Dicer1* in male germ cells results in aberrant meiotic chromosome segregation

Centromeric and pericentric regions have an important role in chromosome segregation during cell division, and transcription of these regions has been implicated to have a functional role in this process (19,20,38). Therefore, we wanted to explore if misregulation of pericentric heterochromatic MSR expression in meiotic spermatocytes in the absence of DICER affects meiotic chromosome segregation. We have earlier shown that deletion of *Dicer1* in early spermatogenic cells mainly affects haploid male germ cell differentiation, causing major defects in chromatin condensation and nuclear shaping of spermatids, which leads to severe oligoasthenoteratozoospermia and infertility (3,4). However, meiotic processes such as synaptonemal complex formation and sex body formation did not show any obvious abnormalities, and meiotic metaphases were normally present at stage XII of the seminiferous epithelial cycle (3,4), suggesting that *Dicer1* cKO germ cells prepare for meiotic divisions normally.

**Figure 7.**
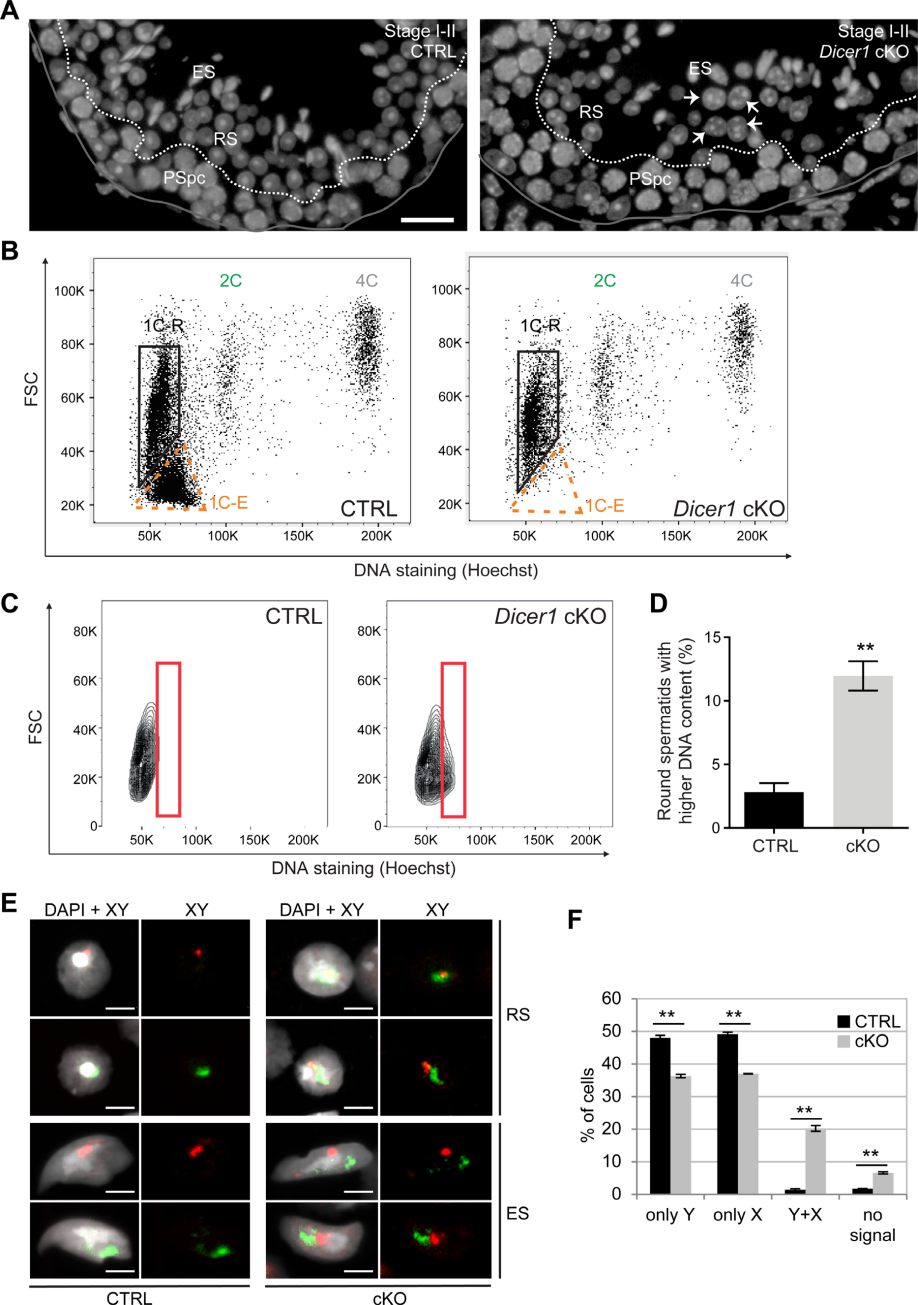
Meiotic chromosome segregation is defective in *Dicer1* knockout germ cells. (**A**) Nuclei of early round spermatids at stage I-II appear unevenly sized in *Dicer1* cKO testes compared to control (CTRL). Nuclei were stained by DAPI (grey). Basal lamina is indicated with a grey line. The border between pachytene spermatocyte (PSpc) and round spermatid (RS) layers is indicated with a dashed white line. ES, elongating spermatids. Some examples of abnormally large spermatid nuclei are indicated with white arrows. Scale bar: 20 μm. (**B**) A representative Hoechst 33342 live-cell fluorescent DNA staining and flow cytometric analysis of DNA ploidy for testicular cells isolated from CTRL and *Dicer1* cKO mice. *Dicer1* cKO testes display the expected 1C, 2C and 4C populations but specifically lack the elongating spermatid population. 1C-R, round spermatids; 1C–E, elongating spermatids; 2C, diploid cells; 4C, tetraploid cells; FSC, forward scatter light. See also Supplementary Figure S6. (**C**, **D**) An in-depth analysis of the 1C-R population reveals that the number of round spermatids having a higher DNA content is significantly higher in *Dicer1* cKO than in CTRL, *n* = 3, SEM, ** *P*< 0.01. (**E**) DNA *in situ* hybridization revealed abnormal number of sex chromosomes in *Dicer1* cKO haploid spermatids. Round spermatid (RS) and elongating spermatid (ES) nuclei containing both X (red) and Y (green) chromosome signals were frequently detected in cKO. Nuclei were stained by DAPI. Scale bar: 5 μm. (**F**) Quantification of the X and Y chromosome signals in *Dicer1* cKO vs. CTRL round spermatids (more than 500 cells per mouse were counted); *n* = 2, SEM, **P< 0.01.

Now we examined the morphology *Dicer1* cKO spermatids in more detail, and observed that early haploid round spermatids right after meiotic division (stage I-II) appeared unevenly sized compared to control round spermatids at the same stage (Figure 7A). Next, we analyzed *Dicer1* cKO testis by flow cytometry. In agreement with previous studies (3,61), we noticed that elongating spermatids were virtually absent from *Dicer1* cKO testis (Figure 7B, Supplementary Figure S6). However, the size of round spermatid population was comparable to that of control testis. Intriguingly, the staining intensity of this population, as judged by DNA dye Hoechst 33342, had shifted slightly but significantly, implicating a higher DNA content in *Dicer1* cKO round spermatids when compared to control (Figure 7C,D). This finding urged us to investigate possible meiotic chromosome mis-segregation and aneuploidy in *Dicer1* cKO testis. Interestingly, *in situ* hybridization using probes detecting sex chromosomes X and Y confirmed an abnormal chromosome number in *Dicer1* cKO round spermatids (Figure 7E,F). We frequently observed spermatids containing both sex chromosomes, indicating defective meiosis I. Altogether these results show that the elevated levels of MSR transcripts in DICER-null spermatocytes resulting from defective MSR processing is accompanied with defects in meiotic chromosome segregation, which significantly contributes to the infertility phenotype of these mice.

## DISCUSSION

Meiotic and postmeiotic male germ cells undergo drastic epigenetic transitions and changes in chromatin organization (62). Meiotic events such as chromosome pairing, synaptonemal complex formation, crossing-over and homologous recombination, are accompanied by active and broad transcription of the genome, producing an unusually diverse transcriptome (2). Here we show that the pericentric heterochromatin is also transcribed in meiotic male germ cells. Furthermore, we elucidate the regulatory mechanisms governing MSR expression by showing that DICER directly associates with MSR transcripts, and inactivation of DICER in germ cells compromises the processing of MSR transcripts into small RNAs. Therefore, DICER function is not restricted to miRNA/siRNA processing during spermatogenesis, but it also participates in the post-transcriptional processing of repeat elements and controls the levels of MSR transcripts.

We have discovered that the forward and reverse strands of MSR have different transcription dynamics, and only the forward MSR transcript expression peaks in pachytene spermatocytes. The forward strand expression has earlier been shown to be prominent in adult mouse testis and liver, but not in other tissues (32,53). Interestingly, the reverse strand has been shown to be present only within seminiferous tubules lacking mature sperm (53), which is in line with our observation that the reverse strand expression appears to be relatively higher in the juvenile testes containing only premeiotic or very early meiotic cells. Temporally tightly controlled expression of forward and reverse strands has been demonstrated during early mouse embryonic development, where the forward strand is expressed only from paternal chromosome at the early two-cell stage of development, while the reverse strand transcription bursts towards the end of the two-cell stage from both maternal and paternal chromosomes (34,35). Depleting either forward or reverse transcript *in vivo* did not affect the levels of the complementary transcript (34), suggesting that their expression is independently regulated. While the regulatory mechanisms of strand-specific transcription have remained obscure, our results clearly show that the differential expression of the forward and reverse strands is evident during male germ cell differentiation.

Furthermore, we show that in the absence of DICER, the level of MSR forward but not reverse transcript is greatly elevated in spermatocytes. The expression analysis during the first wave of spermatogenesis revealed that the difference was most apparent in 14–18 dpp testes, where spermatogenesis has progressed up to pachytene phase of meiosis I. In control mice, the level of MSR forward transcripts fell considerably in 20 dpp testes where the first haploid cells appear, suggesting that early postmeiotic cells have an efficient mechanism to remove MSR transcripts. Interestingly, the postmeiotic drop in MSR forward transcript level was also seen in *Dicer1* cKO testes. Therefore, two distinct mechanisms seem to exist to control the levels of MSR transcripts, a DICER-dependent mechanism in meiotic cells and a DICER-independent mechanism in haploid germ cells.

We also show that DICER forms complexes with MSR forward transcripts *in vivo* in the testis. Further, in the absence of DICER, longer products of MSR forward transcripts accumulate, and the level of MSR transcript-derived small RNAs is reduced. These results strongly support a post-transcriptional role for DICER in processing of MSR transcripts. Similar defects have been reported in cultured somatic cells upon depletion of an acidic nucleoplasmic DNA-binding protein WDHD1 (WD repeat and HMG-box DNA-binding protein 1) that associates with heterochromatin and functions in the post-transcriptional control of both centromeric and pericentric transcripts (63). Interestingly, WDHD1 has been shown to co-operate with DICER, thus linking DICER to MSR processing also in somatic cells (63). DICER has also been implicated in degradation of other repeat transcripts; conditional depletion of DICER in mice induced accumulation of Alu-like B1 and B2 retrotransposon transcripts with a consequence of retinal pigmented epithelium degeneration (64). According to our results, this non-canonical miRNA-independent function of DICER in repeat transcript processing is important for regulation of MSR transcript levels in meiotic spermatocytes.

Increasing evidence supports the functional importance of MSR transcription in the mammalian system. In cell culture models, MSR transcripts have been shown to remain in the pericentric heterochromatin to recruit epigenetic factors, as has been shown for the methyltransferase SUV39H that uses MSR transcripts to stably associate with chromatin (39–41). In mouse, MSR repeat expression was proven important for the *de novo* formation of heterochromatin domains during early embryonic development, and the depletion of MSR transcripts was shown to arrest embryonic development and chromocenter formation (34,35). Interestingly, our results show that the accumulation of MSR transcripts and reduction of MSR-derived small RNAs in *Dicer1* knockout testes is accompanied with a compromised recruitment of histone methyltransferase SUV39H2 on MSR genomic regions, suggesting that DICER-dependent mechanisms are involved in anchoring epigenetic factors on the pericentric heterochromatin in male germ cells.

In addition to the meiotic expression of MSR transcripts shown in this study, they have also been implicated in the regulation of postmeiotic haploid male germ cell differentiation during the time when histones are replaced with protamines (65). This phase of differentiation is characterized by the genome-scale incorporation of histone variants TH2B and H2A.L.2 (66–68). Interestingly, H2A.L.2 was shown to specifically target the pericentric regions of the genome, that was controlled by its ability to bind RNA, therefore highlighting a possible functional role for MSR transcripts in histone incorporation (65). These studies indicate that pericentric regions appear to be privileged sites of histone retention in mature spermatozoa, a process that may have ramifications during early embryonic development.

In this study, we revealed the presence of DICER in the nuclear compartment of spermatocytes. A previous study suggested that the localization of DICER is restricted to the cytoplasm during mouse spermatogenesis (69). However, this analysis was done using genetically-modified mice expressing HA-tagged DICER, which may be the reason for the discrepancies in terms of nuclear localization. Importantly, we revealed that DICER is associated with chromatin and specifically with genomic MSR sequences, and it interacts with heterochromatin regulators such as TRIM28, TRIM33 and histone methyltransferases. These data suggest that DICER is recruited to the site of MSR transcription by heterochromatin proteins, and it likely regulates heterochromatin properties in mammalian germ cells in an analogous way to lower organisms that utilize the chromatin-associated DICER to control heterochromatin formation and maintenance (33,70,71). However, our study did not specifically address the role of cytoplasmic vs. nuclear DICER in the processing of MSR transcripts, and it is possible that some steps take place in the cytoplasm. Despite the uncertainty about the exact subcellular site of DICER action, the functional consequences of DICER ablation are obvious: defective processing of MSR transcripts, reduced recruitment of SUV39H2 and H3K9me3 to pericentric heterochromatin and infidelity of meiotic chromosome segregation.

The inactivation of DICER-dependent activities is deleterious for male germ cell differentiation. *Dicer1* cKO males are infertile with disrupted haploid differentiation process accompanied with severe defects in cell polarization, acrosomal formation, nuclear elongation and chromatin condensation (3,4). Although meiosis progresses in the absence of DICER to produce haploid round spermatids, we demonstrate here that a considerable proportion of *Dicer1* cKO haploid cells have an abnormal DNA content and display aneuploidy, and therefore it is evident that at least a part of observed haploid problems originate from errors in chromosomal segregation. Whether MSR transcripts and the derivative small RNAs have a direct role in meiotic chromosomal segregation is an intriguing question. However, it is challenging to address using a physiological model, such as the *Dicer1* cKO mouse model due to the difficulties in dissecting MSR-specific defects from the defects originating from other DICER-dependent processes (e.g. miRNA processing). Nonetheless, given the importance of pericentric and centromeric heterochromatin in chromatin organization and chromosome segregation (19,20,24–28,38), it is a plausible hypothesis that the defects in the control of MSR transcript expression and processing contribute to the meiotic phenotype of *Dicer1* cKO mice.

In summary, we have revealed a novel physiological function for DICER in male germ cells by showing that it represses MSR transcript levels during spermatogenesis. We demonstrate that DICER associates with both MSR genomic regions and MSR transcripts, and the elevated level of MSR transcripts in *Dicer1* cKO testis originates from their defective processing. Furthermore, we provide evidence that these defects impinge on the epigenetic status of pericentric heterochromatin and the fidelity of meiotic chromosome segregation thus compromising the integrity of the germline. Altogether, our results suggest that the function of DICER in control of pericentric heterochromatin expression significantly contributes to the production of fertile spermatozoa, and therefore maintenance of male fertility.

## DATA AVAILABILITY

The mass spectrometry proteomics data have been deposited to the ProteomeXchange Consortium via the PRIDE (72) partner repository with the dataset identifier PXD017772.

## Supplementary Material

gkaa460_Supplemental_Files
