# Supplementary material for: DICER regulates the expression of major satellite repeat transcripts and meiotic chromosome segregation during spermatogenesis
Source: Nucleic Acids Res. 2020 Jun 2;48(13):7135–53. doi: 10.1093/nar/gkaa460 (PMC7367195; doi:10.1093/nar/gkaa460)
Supplement: gkaa460_Supplemental_Files [file gkaa460_supplemental_files.zip › Yadav_supplementary_material.pdf]

SUPPLEMENTARY MATERIAL

DICER regulates the expression of major satellite repeat transcripts and meiotic chromosome segregation during spermatogenesis

Ram Prakash Yadav, Juho-Antti Mäkelä, Hanna Hyssälä, Sheyla Cisneros-Montalvo, Noora Kotaja

Supplementary Table

Table S1. Mass spectrometry of DICER-interacting proteins in 17-18 dpp mouse testes. Related to Figure 4 and Supplementary Figure S5.

Supplementary Videos

Video S1. 3D model of the DICER localization in a spermatocyte. Related to Figure 5. A testis section was stained with anti-DICER (green) and DAPI (violet). The Z-stack was captured by laser scanning confocal microscope (Zeiss LSM780), and the 3D model was constructed and video was developed by ZEN 2.5 lite software (Carl Zeiss Microscopy GmbH, Germany). The video was converted to mp4 format using ANIMOTICA (version: v1.1.87.0).

Video S2. 3D model of the association of DICER nuclear foci with heterochromatin in a spermatocyte. Related to Figure 5. A testis section was stained with anti-DICER (red), anti-HP1β (green) and DAPI (grey). The Z-stack was captured by laser scanning confocal microscope (Zeiss LSM780), and the 3D model was constructed and video was developed by ZEN 2.5 lite software (Carl Zeiss Microscopy GmbH), Germany. The video was converted to mp4 format using ANIMOTICA (version: v1.1.87.0).

Supplementary Figures S1-S6

Figure S1

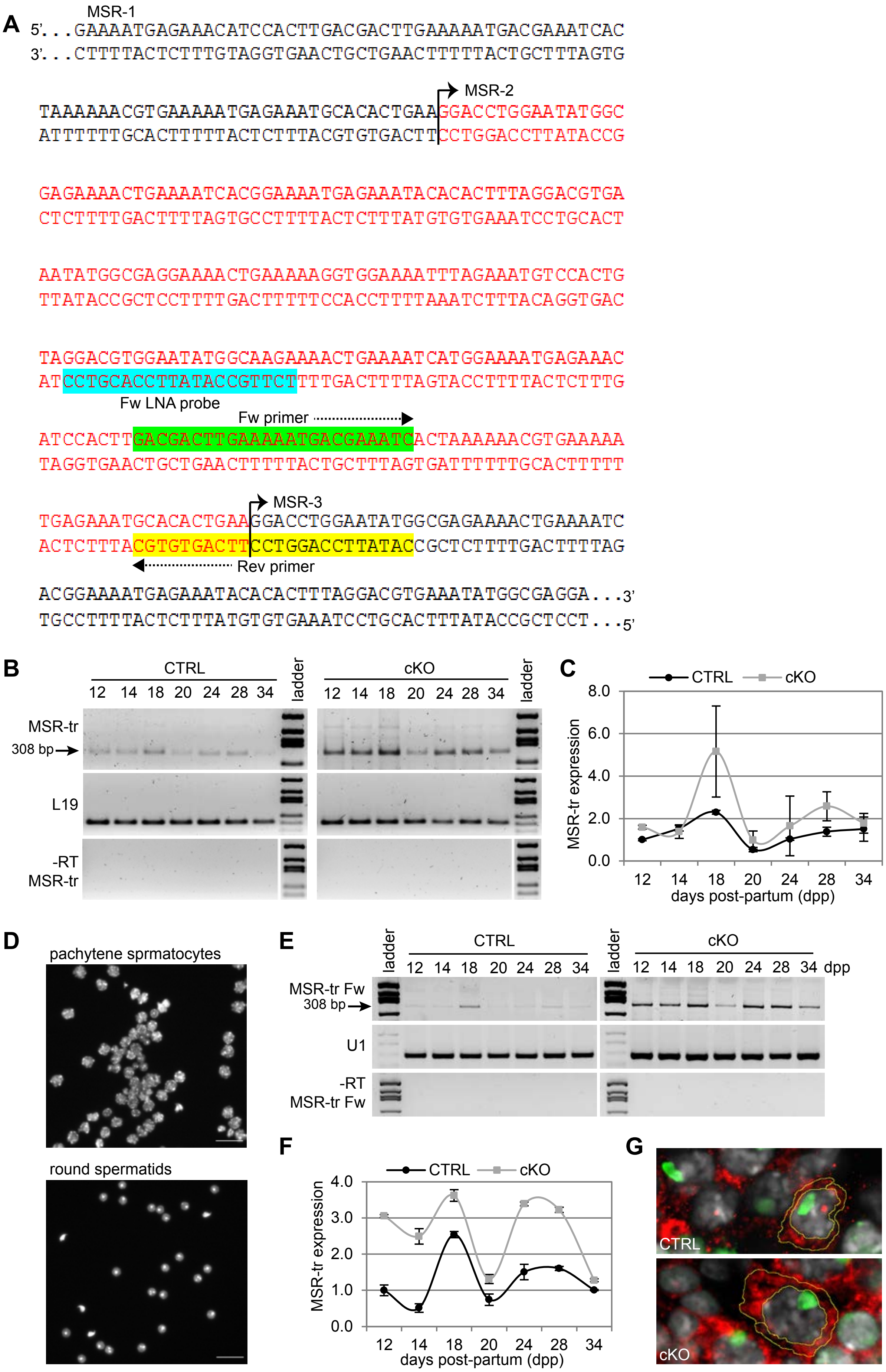
A
MSR-1
5'...GAAAATGAGAAACATCCACTTGACGACTTGAAAAATGACGAAATCAC
3'...CTTTTACTCTTTGTAGGTGAACTGCTGAACTTTTTACTGCTTTAGTG
MSR-2
TAAAAAACGTGAAAAATGAGAAATGCACACTGAAGGACCTGGAATATGGC
ATTTTTTGCACTTTTTACTCTTTACGTGTGACTTCCTGGACCTTATACCG
GAGAAAACTGAAAATCACGGAAAATGAGAAATACACACTTTAGGACGTGA
CTCTTTTGACTTTTAGTGCCTTTTACTCTTTATGTGTGAAATCCTGCACT
AATATGGCGAGGAAAACTGAAAAAGGTGGAAAATTTAGAAATGTCCACTG
TTATACCGCTCCTTTTGACTTTTTCCACCTTTTAAATCTTTACAGGTGAC
TAGGACGTGGAATATGGCAAGAAAACTGAAAATCATGGAAAATGAGAAAC
ATCCTGCACCTTATACCGTTCTTTTGACTTTTAGTACCTTTTACTCTTTG
Fw LNA probe
Fw primer
ATCCACTTGACGACTTGAAAAATGACGAAATCACTAAAAAACGTGAAAAA
TAGGTGAACTGCTGAACTTTTTACTGCTTTAGTGATTTTTTGCACTTTTT
MSR-3
TGAGAAATGCACACTGAAGGACCTGGAATATGGCGAGAAAACTGAAAATC
ACTCTTTACGTGTGACTTCCTGGACCTTATACCGCTCTTTTGACTTTTAG
Rev primer
ACGGAAAATGAGAAATACACACTTTAGGACGTGAAATATGGCGAGGA...3'
TGCCTTTTACTCTTTATGTGTGAAATCCTGCACTTTATACCGCTCCT...5'
B
CTRL
cKO
12 14 18 20 24 28 34
ladder
MSR-tr
308 bp
L19
-RT MSR-tr
C
CTRL
cKO
8.0
6.0
4.0
2.0
0.0
MSR-tr expression
12 14 18 20 24 28 34
days post-partum (dpp)
D
pachytene sprmatocytes
round spermatids
E
ladder
CTRL
cKO
12 14 18 20 24 28 34 dpp
MSR-tr Fw
308 bp
U1
-RT MSR-tr Fw
F
CTRL
cKO
4.0
3.0
2.0
1.0
0.0
MSR-tr expression
12 14 18 20 24 28 34
days post-partum (dpp)
G
CTRL
cKO

Figure S1. Expression of major satellite repeats in *Dicer1* cKO testis during the first wave of spermatogenesis. Related to Figures 1, 2 and 4. (**A**) MSR consensus sequence. One consensus repeat (labelled as MSR-2) is shown in whole (red). The positions of the forward (Fw, green highlight) and reverse (Rev, yellow highlight) primers used for PCR and strand-specific cDNA amplification, as well as the forward strand-detecting LNA probe (cyan highlight) are indicated. (**B**) RT-PCR analysis to detect MSR transcript (MSR-tr) expression during the first wave of spermatogenesis in control (CTRL) vs. *Dicer1* knockout (cKO) mice. Dpp, days post-partum. *L19* was used as a reference gene. The reactions without a reverse transcriptase enzyme (-RT) control genomic DNA contamination. (**C**) The intensity of 308 bp MSR-tr bands was quantified using Image J and normalized to the signal intensity of the reference gene band in the same sample. MSR-tr expression is shown relative to the CTRL 12 dpp sample that was set as ‘1’. The level of MSR-tr was elevated in *Dicer1* cKO testes compared to the control, particularly at 18 dpp. n=2, SEM. (**D**) DAPI-stained pachytene spermatocyte and round spermatid fractions enriched by centrifugal elutriation. Both fractions were evaluated to be >85% pure. Scale bars: 25 µm. (**E**) Control and *Dicer1* cKO testis samples were collected at different time points during the first wave of spermatogenesis, and MSR forward strand expression was analyzed by semi-quantitative RT-PCR. The forward strand transcript levels were generally higher in *Dicer1* cKO testes. (**F**) Quantification of the intensity of 308 bp MSR-tr Fw bands by ImageJ. The values were normalized to the reference gene expression in the same sample. MSR-tr Fw expression is shown relative to CTRL Fw 12 dpp sample that was set as ‘1’. (**G**) Visualization of the cytoplasmic area selected for the quantification of average signal intensity of MSR forward transcript signal in Figure 4C.

Figure S2

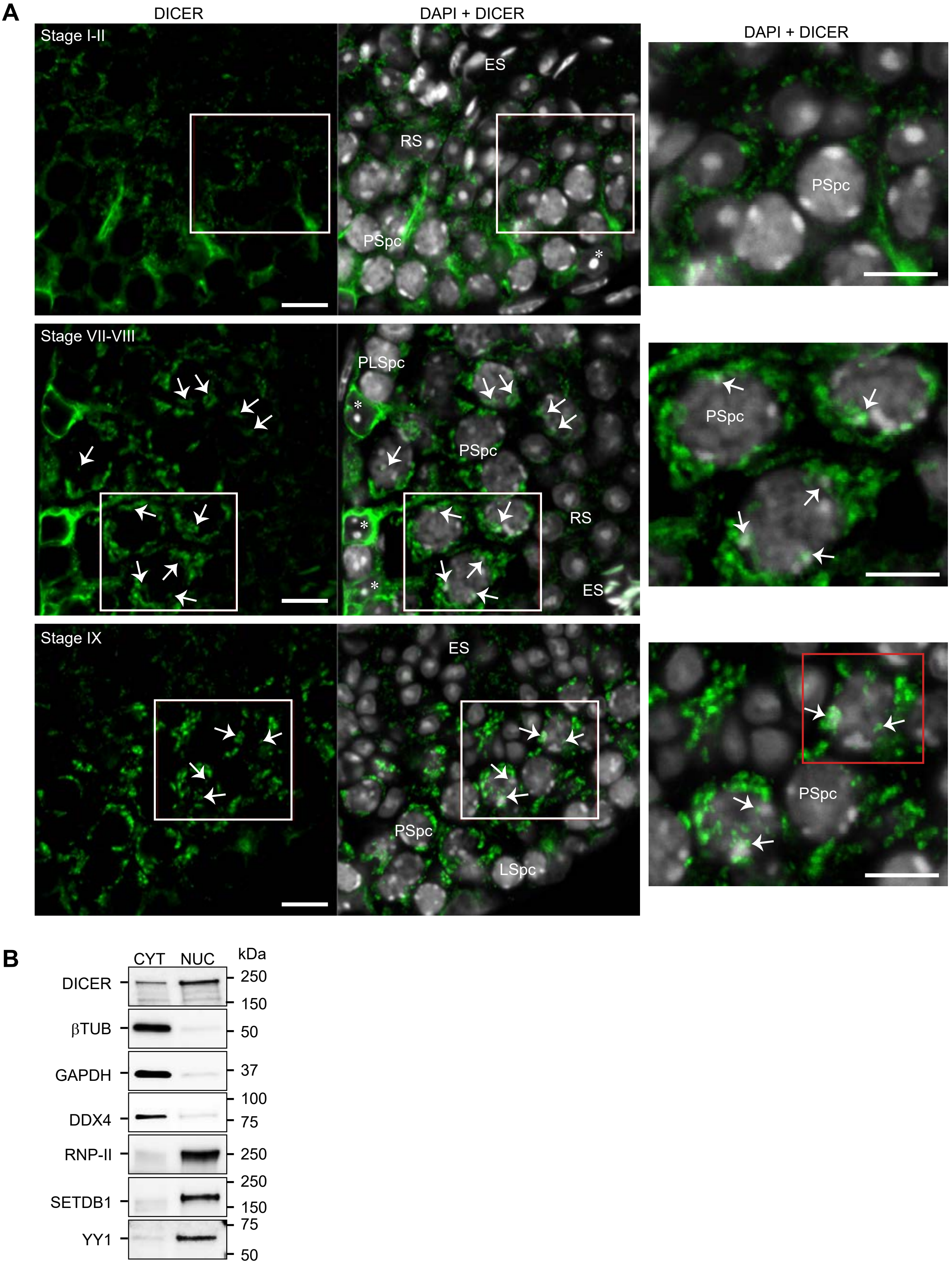
A
DICER
DAPI + DICER
DAPI + DICER
Stage I-II
ES
RS
PSpc
PSpc
Stage VII-VIII
PLSpc
PSpc
RS
ES
PSpc
Stage IX
ES
PSpc
LSpc
PSpc
B
CYT
NUC
kDa
DICER
βTUB
GAPDH
DDX4
RNP-II
SETDB1
YY1
250
150
50
37
100
75
250
250
150
75
50

Figure S2. Localization of DICER in the seminiferous epithelium. Related to Figure 5. (**A**) PFA-fixed paraffin-embedded WT adult mouse testis sections were immunostained using an anti-DICER antibody (green). Nuclei were stained by DAPI (grey). Images show DICER localization at stages I-II, VII-VIII and IX of the seminiferous epithelial cycle. PLSpc, preleptotene spermatocyte; LSpc, leptotene spermatocyte; PSpc, pachytene spermatocyte; RS, round spermatid; ES, elongating spermatid. Asterisks indicate Sertoli cell nuclei. Some examples of DICER-positive nuclear foci are pointed by white arrows. The areas indicated with white boxes are shown with higher magnification in the right panel. The cell indicated with a red box is shown in **Figure 5A** and in **Supplementary Video S1**. Scale bar: 10 µm. (**B**) Immunoblotting of cytoplasmic (CYT) and nuclear (NUC) extracts from 16-18 dpp mouse testicular cells revealed the presence of DICER in both fractions. Antibodies against cytoplasmic proteins β-TUBULIN, GAPDH (Glyceraldehyde-3-phosphate dehydrogenase) and DDX4 (DEAD-Box Helicase 4), as well as nuclear proteins RNA polymerase II, (SET domain bifurcated histone lysine methyltransferase 1) and YY1 (Yin Yang 1, a transcriptional repressor) were included to validate the purity of the extracts. Note that nuclear samples are more concentrated, which hinders the direct comparison of protein levels between the nuclear and cytoplasmic fractions. The experiment was independently repeated two times.

Figure S3

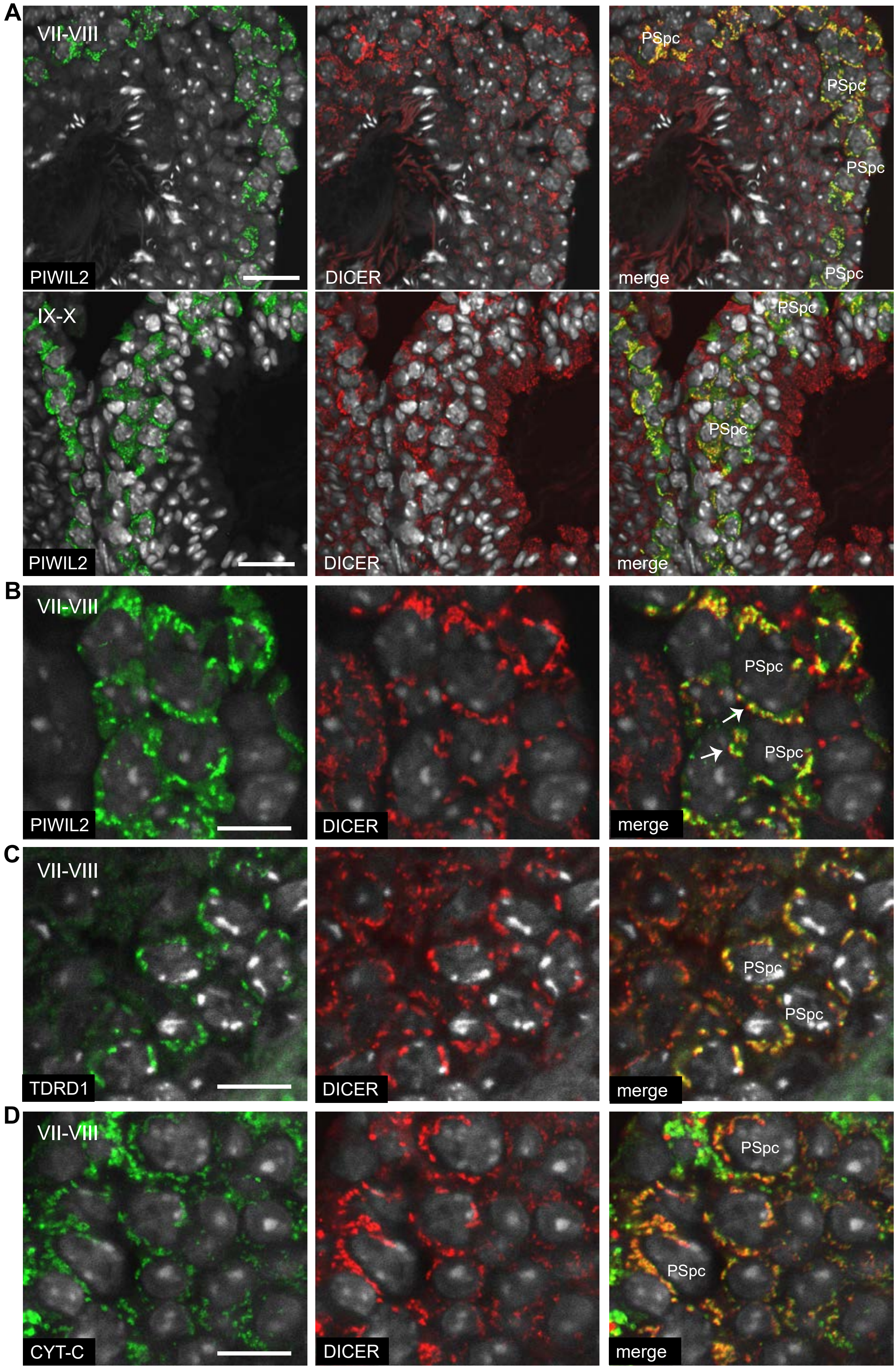
A
VII-VIII
PIWIL2
DICER
merge
PSpc
PSpc
PSpc
PSpc
IX-X
PIWIL2
DICER
merge
PSpc
PSpc
B
VII-VIII
PIWIL2
DICER
merge
PSpc
PSpc
C
VII-VIII
TDRD1
DICER
merge
PSpc
PSpc
D
VII-VIII
CYT-C
DICER
merge
PSpc
PSpc

Figure S3. DICER localizes to the intermitochondrial cement (IMC) in pachytene spermatocytes. Related to Figure 5. (**A**) Co-immunostaining of PFA-fixed paraffin-embedded WT adult mouse testis sections using anti-PIWIL2/MILI (green) and anti-DICER (red) antibodies demonstrated the co-localization of PIWIL2 and DICER in the IMC of mid-pachytene spermatocytes at stage VIII and late-pachytene spermatocytes at stage X of the seminiferous epithelial cycle. Nuclei were stained by DAPI (grey). Scale bar: 25 µm. (**B**) Higher magnification images of the co-immunostainings with anti-PIWIL2 and anti-DICER revealed that DICER signal was found in the same granular structures as PIWIL2, either completely overlapping (yellow), or adjacent to the PIWIL2 granules (red, examples indicated with arrows). (**C**),(**D**) Localization of DICER to the IMC was further validated by co-immunostaining with anti-TDRD1 and anti-Cytochrome C antibodies, respectively. PSpc: pachytene spermatocyte. Scale bar for **B-D**: 10 µm. Panels show a representative figure of an experiment that was independently repeated several times.

Figure S4

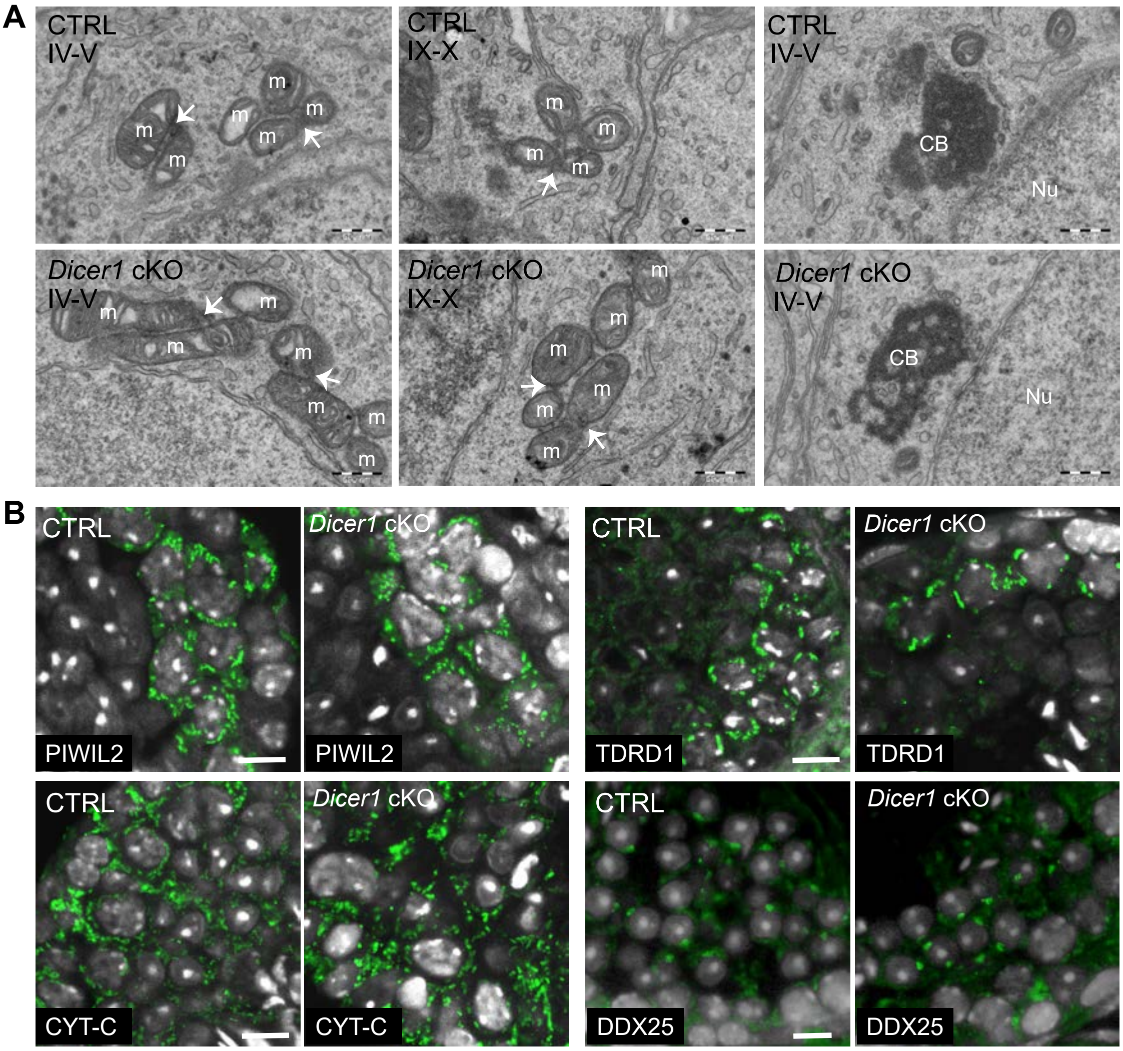
A
CTRL
IV-V
m
CTRL
IX-X
m
CTRL
IV-V
CB
Nu
Dicer1 cKO
IV-V
m
Dicer1 cKO
IX-X
m
Dicer1 cKO
IV-V
CB
Nu
B
CTRL
PIWIL2
Dicer1 cKO
PIWIL2
CTRL
TDRD1
Dicer1 cKO
TDRD1
CTRL
CYT-C
Dicer1 cKO
CYT-C
CTRL
DDX25
Dicer1 cKO
DDX25

Figure S4. The morphology of the IMC and CB is not affected in *Dicer1* knockout germ cells. Related to Figure 5. (**A**) The morphology of IMC (arrows) between the mitochondrial (m) clusters in early (stage IV-V) and late (stage IX-X) pachytene spermatocytes, and the CB in round spermatids (stage IV-V) appeared unaffected in *Dicer1* knockout (cKO) mice in electron microscopic analysis. Nu: nucleus. Scale bars: 500 nm. (**B**) Immunostaining of control (CTRL) and *Dicer1* cKO testis sections showed comparable localization of PIWIL2, TDRD1 and Cytochrome C in pachytene spermatocytes at stage VII-VIII (confocal images). DDX25 localization in the CB of round spermatids at stage II-V was not affected in the absence of DICER (widefield fluorescence images). Scale bars: 10 μm. The immunostaining was independently repeated using at least two biological replicates.

Figure S5

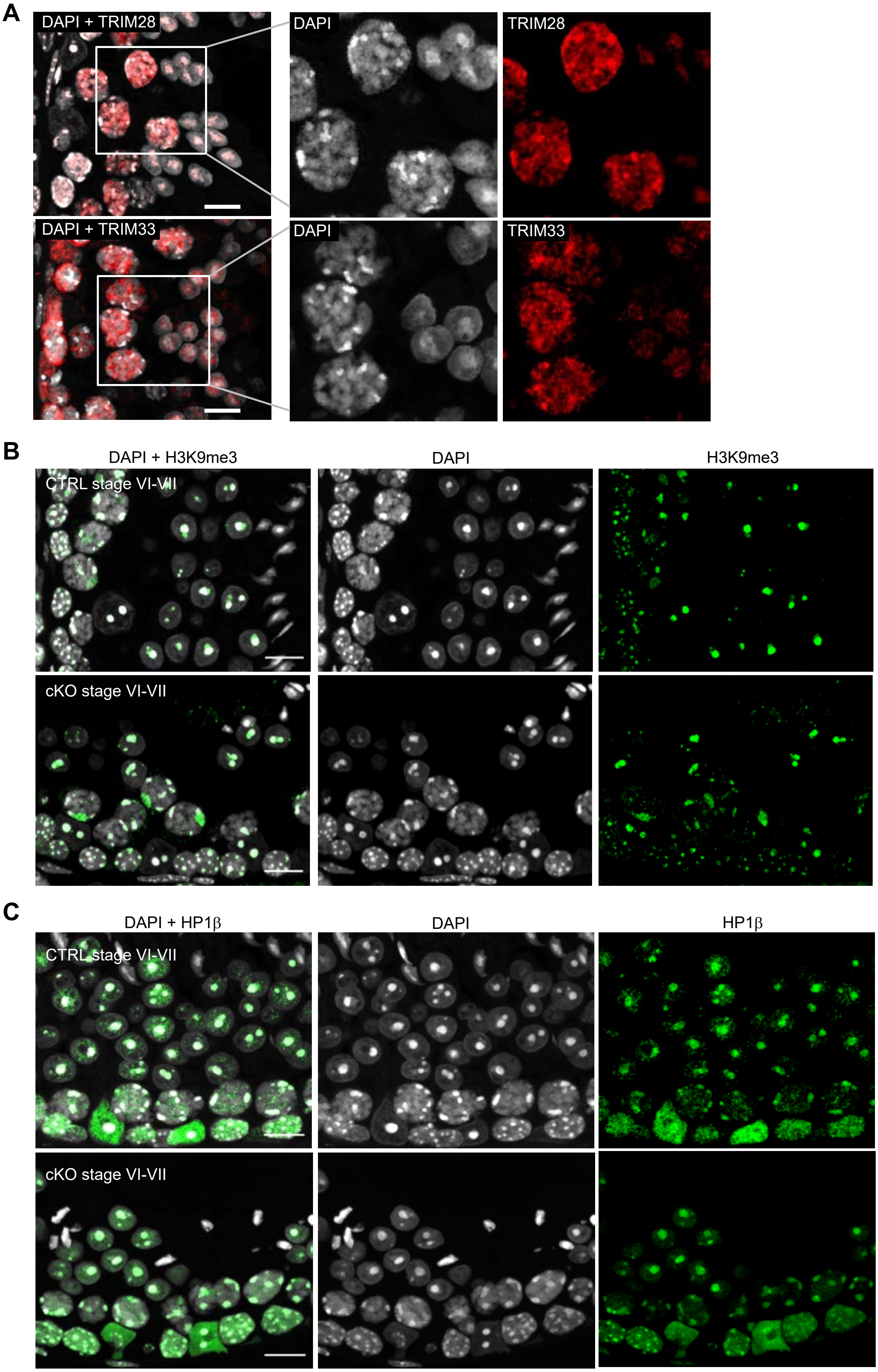
A
DAPI + TRIM28
DAPI
TRIM28
DAPI + TRIM33
DAPI
TRIM33
B
DAPI + H3K9me3
DAPI
H3K9me3
CTRL stage VI-VII
cKO stage VI-VII
C
DAPI + HP1β
DAPI
HP1β
CTRL stage VI-VII
cKO stage VI-VII

Figure S5. Localization of TRIM28 and TRIM33 in wild type testes and the localization of heterochromatin markers in *Dicer1* cKO testes. Related to Figure 6 and Table S1. (**A**) TRIM28 and TRIM33 localize to the nucleus of male germ cells (stage IX-X). Immunofluorescence was performed on adult paraffin-embedded testis sections using antibodies against TRIM28 and TRIM33 (red). Nuclei were stained with DAPI (grey). Higher magnification images on the right panel are from the areas indicated by white boxes. Scale bar: 10 µm. (**B**),(**C**) Localization of heterochromatin markers appears not to be affected in *Dicer1* cKO testes. PFA-fixed paraffin-embedded CTRL and *Dicer1* cKO testis sections were immunostained with antibodies against H3K9me3 (**B**) or HP1β (**C**). Nuclei were stained by DAPI. Stage VI-VII of the seminiferous epithelial cycle is shown for both markers. Both heterochromatin markers were found to be normally associated with DAPI-bright chromocenters. Scale bar: 10 µm. Panels show a representative figure of an experiment that was independently repeated with at least two biological replicates.

Figure S6

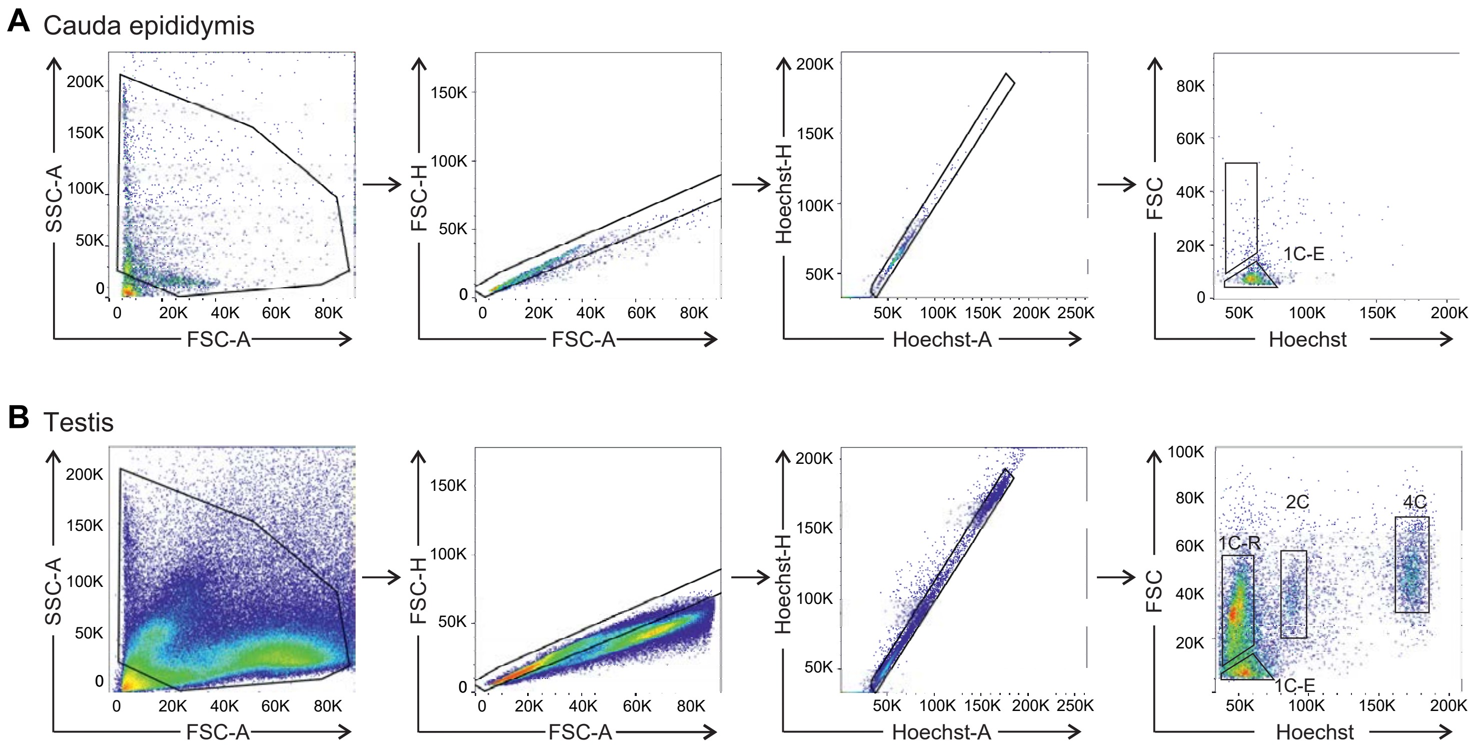
A
Cauda epididymis
SSC-A
FSC-A
FSC-H
FSC-A
Hoechst-H
Hoechst-A
FSC
Hoechst
1C-E
B
Testis
SSC-A
FSC-A
FSC-H
FSC-A
Hoechst-H
Hoechst-A
FSC
Hoechst
1C-R
2C
4C
1C-E

Figure S6. Live-cell fluorescent DNA staining and flow cytometric analysis of DNA ploidy. Related to Figure 7. Flow cytometric analysis was performed for cells isolated from WT cauda epididymis (**A**) and testis (**B**)**.** Enrichment of elongating spermatids is observed in the cauda epididymis. These data are used as a gating strategy and they indicate that elongated spermatids (1C-E) constitute a separate population within the 1C subset in flow cytometric analysis of testicular cells. 1C-R, round spermatids; 2C, diploid cells; 4C, tetraploid cells.
